# Immunoinformatics-Driven Rational Design of Altered Peptide Ligands and In Vitro Validation of a Multi-Epitope CTL Formulation Targeting HPV16 E6/E7

**DOI:** 10.3390/vaccines14080715

**Published:** 2026-08-20

**Authors:** Dian Dong, Xiuqing Zhang, Bo Li

**Affiliations:** 1 College of Life Sciences, University of Chinese Academy of Sciences, Beijing 100049, China; dongdian19@mails.ucas.ac.cn (D.D.); zhangxq@genomics.cn (X.Z.); 2 BGI Research, Shenzhen 518083, China

**Keywords:** HPV16, E6/E7, altered peptide ligand, HLA-A*02:01, cytotoxic T lymphocytes, peptide-based immunotherapy, immunoinformatics, molecular dynamics, mRNA vaccine

## Abstract

**Background**: Therapeutic vaccination against human papillomavirus type 16 (HPV16) is frequently limited by the weak HLA-A*02:01 presentation of native E6/E7 oncoprotein epitopes. This study aims to utilize an integrated computational-experimental pipeline to design and evaluate anchor-optimized altered peptide ligands (APLs) for improving peptide presentation and HPV16-specific T-cell responses. **Methods**: Anchor-residue substitutions were introduced into three wild-type E6/E7 epitopes. Candidates were prioritized in silico based on predicted presentation, affinity, immunogenicity, and toxicity, and subsequently evaluated via in vitro functional assays using HLA-A*02:01-positive donor cells and molecular dynamics (MD) simulations. **Results**: Anchor optimization successfully converted weak binders into strong binders; for instance, E6apl improved predicted affinity from 329.33 to 6.21 nM. Crucially, candidate E7apl1 exhibited normal CD8^+^ T-cell expansion but reduced IFN-γ secretion, revealing a distinct binding–immunogenicity dissociation. MD simulations suggested that altered peptide conformational dynamics may contribute to differences in functional activity. Subsequently, an optimized six-peptide formulation (three APLs and three wild-type epitopes) was assembled. The resulting multi-epitope HPV-specific cytotoxic T lymphocytes (meHPV-CTLs) mediated target-specific cytotoxicity against cervical cancer cells, achieving 74.1% ± 5.1% specific lysis at an effector-to-target ratio of 30:1, which was largely abrogated by HLA class I blockade. **Conclusions**: These proof-of-concept findings demonstrate that stable peptide–MHC binding is a necessary but insufficient condition for optimal T-cell activation. The experimentally characterized multi-epitope formulation provides a proof-of-concept strategy for further preclinical evaluation of HPV16-targeted peptide-based immunotherapies. Moreover, because these anchor-optimized APLs are defined at the sequence level, these sequence-defined APLs may potentially be explored in alternative vaccine delivery platforms, including mRNA-based approaches, in future studies.

## 1. Introduction

Persistent infection with high-risk human papillomavirus type 16 (HPV16) is the principal cause of cervical cancer and a substantial fraction of other anogenital and oropharyngeal malignancies, imposing a major global health burden [1]. Although prophylactic vaccines have significantly reduced the incidence of new HPV infections, they do not eliminate established infections or treat existing malignancies, leaving a large population of patients with HPV16-associated disease without effective antigen-specific therapeutic options [2]. The constitutive expression of the E6 and E7 oncoproteins in transformed cells, their essential roles in malignant progression, and their absence from normal tissues make them attractive tumor-specific antigens for therapeutic vaccination strategies aimed at eliciting cytotoxic T-lymphocyte (CTL) responses [3]. Accordingly, the identification of immunogenic HPV16 E6/E7 epitopes capable of inducing durable CD8^+^ T-cell responses remains a central objective in therapeutic vaccine development [4,5].

A major challenge in this context is that many native HPV16 E6/E7 epitopes are presented inefficiently by common HLA class I alleles, including the prevalent HLA-A*02:01 [6]. Effective CD8^+^ T-cell priming requires stable peptide–major histocompatibility complex (pMHC) interactions, and accumulating evidence suggests that the durability and quality of CTL responses correlate more closely with pMHC stability than with equilibrium binding affinity alone [7]. Native E6/E7 epitopes frequently contain suboptimal residues at key HLA-A*02:01 anchor positions, resulting in reduced peptide presentation and limited immunogenicity [8]. Improving peptide presentation is therefore an important prerequisite for E6/E7-directed therapeutic vaccination, although enhanced HLA binding alone may not be sufficient to ensure optimal downstream T-cell function.

Altered peptide ligands (APLs), also termed heteroclitic or anchor-modified peptides, represent one strategy to overcome this limitation by introducing substitutions at buried HLA anchor positions while preserving residues involved in T-cell receptor (TCR) recognition [9]. Such modifications have been shown to improve HLA-A2 binding and modulate antigen-specific T-cell responses in multiple tumor and viral antigen systems [10]. However, improvements in predicted peptide–HLA binding do not invariably translate into enhanced functional T-cell responses. Consequently, candidate epitopes must be evaluated not only for their predicted presentation characteristics but also for their ability to support downstream T-cell activation and effector function [11]. Immunoinformatics approaches enable efficient prioritization of candidate epitopes according to predicted presentation, immunogenicity, antigenicity, and toxicity, whereas molecular dynamics (MD) simulations and MM-GBSA analyses can provide complementary structural insight into how anchor substitutions influence interaction properties at the atomic level [12]. Beyond affinity-based predictors such as NetMHCpan, emerging Transformer-based deep-learning models (e.g., TransHLA) enable direct detection of HLA-presented epitopes and may further complement candidate epitope prioritization [13]. Integrating computational prediction with functional evaluation is therefore essential for the rational development of multi-epitope peptide-based immunotherapeutic strategies.

In this study, we combined immunoinformatics-guided epitope engineering with experimental evaluation to design HLA-A*02:01-restricted altered peptide ligands targeting HPV16 E6 and E7. Candidate peptides were generated through anchor-residue optimization and prioritized using predicted presentation, immunogenicity, antigenicity, and toxicity metrics. To determine whether computationally selected epitopes could support functional T-cell responses, the prioritized peptides were evaluated for CD8^+^ T-cell expansion, effector function, HLA binding, and cytotoxic activity in HLA-A*02:01-positive donor cells. Based on sequential computational and functional screening, a six-peptide formulation comprising three wild-type epitopes and three optimized APLs was assembled and used to generate multi-epitope HPV-specific cytotoxic T lymphocytes (meHPV-CTLs). In parallel, MD simulations, MM-GBSA calculations, and COCOMAPS 2.0 analyses were performed to further characterize how anchor-residue substitutions influenced peptide–HLA interaction patterns and structural properties. Overall, this work provides an integrated computational-to-functional framework for evaluating HPV16 E6/E7-derived APL candidates and offers structural and experimental insights to guide further preclinical investigation of meHPV-CTL-based peptide immunotherapeutic strategies.

## 2. Materials and Methods

### 2.1. Peptides, Cell Lines, HLA Typing, and Ethics

Peptides. Peptides were custom-synthesized by Anhui Guotai Biotechnology Co., Ltd. (Xuancheng, Anhui, China) and Anhui Huajun Pharmaceutical Co., Ltd. (Fuyang, Anhui, China); purity exceeded 98% as determined by HPLC and peptide identity was confirmed by mass spectrometry. For cellular assays, peptides were dissolved in dimethyl sulfoxide (DMSO; Origen Biomedical, Austin, TX, USA) as a concentrated stock and diluted in the relevant culture medium immediately before use, with the final DMSO concentration kept below 1% (*v*/*v*).

Cell lines. The parental SiHa cervical carcinoma line (HLA-A*24:02, mismatched to the HLA-A*02:01 restriction of the peptides, with low endogenous HPV16 E7) was lentivirally transduced to co-express HLA-A*02:01 and HPV16 E7 and was antibiotic-selected, yielding the HLA-matched, E7-enhanced target SiHa-0201-E7. Unmodified parental SiHa was retained as an HLA-mismatched, low-antigen control target. TAP-deficient T2 cells (174 × CEM.T2; HLA-A*02:01-positive; in-house cell bank) [14] were maintained in IMDM (Gibco/Thermo Fisher Scientific, Waltham, MA, USA) supplemented with 10% fetal bovine serum (ExCell Bio, Shanghai, China); because T2 cells lack functional TAP, surface HLA-A*02:01 is expressed at low levels and is stabilized only upon binding of exogenously supplied peptide [15]. Peripheral blood mononuclear cells (PBMCs) were isolated from HLA-A*02:01-positive healthy donors by density-gradient centrifugation and were the source of CD8^+^ T cells and monocyte-derived dendritic cells (DCs). Routine consumables included phosphate-buffered saline (PBS; Gibco) and standard tissue-culture plasticware (Corning, Corning, NY, USA).

HLA typing. Donor HLA-A*02:01 status was determined by whole-exome sequencing-based HLA typing: HLA-region reads were extracted with POLYSOLVER (v1.0) [16] and four-digit typing was performed with OptiType (v1.3.2) [17]. The three healthy donors used in the functional assays (HD1–HD3) were confirmed to be HLA-A*02:01-positive.

Ethics. The study was approved by the Institutional Review Board of BGI (protocol code BGI-IRB 23023, approved 17 January 2023), and written informed consent was obtained from all donors.

### 2.2. In Silico Prediction of Epitopes and APLs

The full-length amino acid sequences of the HPV16 E6 (158 aa) and E7 (98 aa) oncoproteins were retrieved from the HPV16 reference genome (Seedorf et al. [18]; NCBI Reference Sequence NC_001526 [19]). Overlapping 8–11mer peptides spanning each protein were enumerated using a sliding-window approach. HLA-A*02:01-restricted presentation was predicted with NetMHCpan-4.1 [20], and candidates were cross-referenced against experimentally validated epitopes curated in the Immune Epitope Database (IEDB) [21] and against clinical evidence from structured literature mining. Wild-type (WT) epitopes were prioritized when they satisfied at least two of three independent criteria (predicted natural presentation, prior evidence of immunogenicity, and reported clinical relevance), yielding three lead WT peptides: E6wt (TIHDIILECV), E7wt1 (LLMGTLGIV), and E7wt2 (YMLDLQPETT).

Altered peptide ligands (APLs) were generated from the three lead epitopes by canonical HLA-A*02:01 anchor optimization, introducing substitutions at P1 (→Y), P2 (→L), and the C-terminal anchor (→V), singly or in combination, conditional on the native residue. This procedure produced seven candidate APLs, which were then subjected to the computational filters described below.

The seven candidates were filtered by predicted HLA-A*02:01 binding (NetMHCpan-4.1 [20]; %Rank ≤ 0.5% defining a strong binder), intrinsic immunogenicity (IEDB Class I Immunogenicity Predictor [22]), predicted non-toxicity (ToxinPred [23]), and whole-peptide antigenicity (VaxiJen 3.0 [24,25]; threshold > 0.4), followed by expert manual curation. All computational analyses were performed for HLA-A*02:01 using the native peptide lengths (9- or 10-mer). Residue numbering refers to positions within the mature HPV16 E6 and E7 proteins. Candidates passing all predefined criteria were advanced, together with their parental wild-type epitopes, to downstream experimental validation.

### 2.3. T2 Peptide-Binding Assay

Peptide binding to HLA-A*02:01 was measured by T2-cell surface stabilization. T2 cells were seeded at 5 × 10^5^ cells per well in serum-free IMDM, pulsed overnight (~16–18 h, 37 °C, 5% CO_2_) with each peptide at 100 µg/mL; no exogenous β_2_-microglobulin was added. Unloaded T2 cells were the baseline control, and a TWISTNB-derived HLA-A*02:01-restricted neoantigen peptide (KLMGIVYKV) served as a positive control. Cells were stained for surface HLA-A2 with an APC-conjugated anti-human HLA-A2 antibody (clone BB7.2; BioLegend, San Diego, CA, USA; Cat. 343308) [26], acquired on a CytoFLEX flow cytometer (Beckman Coulter, Brea, CA, USA), and analyzed with FlowJo software (v10.8.1; BD Biosciences, Ashland, OR, USA). Surface HLA-A2 was quantified as the APC mean fluorescence intensity (MFI), and binding was expressed as the MFI enhancement index [(MFI_peptide_ − MFI_unloaded)_/MFI_unloaded_], with an index ≥ 1 considered indicative of strong binding. The assay was performed in duplicate (*n* = 2) and reported as the arithmetic mean.

The E7wt1 and E7apl1 peptides were not evaluated in T2 assay because E7wt1 required DMSO at concentrations cytotoxic to T2 cells; E7apl1 binding is instead supported by the in silico prediction (Table 1) and the MD analysis (Table 2), and was therefore not subjected to experimental T2 validation.

### 2.4. In Vitro T-Cell Expansion (meHPV-CTL Generation) and IFN-γ ELISpot Assay

CD8^+^ T cells were purified from PBMCs by magnetic-activated cell sorting, and monocyte-derived DCs were generated in DC culture medium (CellGenix, Freiburg, Germany). DCs were pulsed individually with each peptide (5 µg/mL, 3.5 h). CD8^+^ T cells were stimulated over three sequential 7–9-day rounds with autologous peptide-pulsed DCs (DC:T-cell ratio 1:4 for round 1 and 1:8 thereafter) in HIPP-T009 serum-free lymphocyte medium (BeiNuoJi Shanghai, China) supplemented with autologous plasma (2.5%, *v*/*v*) and the interleukins IL-21, IL-7, IL-15, and IL-2 each at 10 ng/mL (all from Shanghai Puxin, Shanghai, China); fold expansion per round was calculated as harvested viable cells divided by input cells. Viability and concentration were determined on a Countstar Rigel S2 fluorescence cell analyzer (Countstar, Shanghai, China) by acridine orange/propidium iodide staining. Third-round CTLs (CTL3) were used for downstream assays. Cultures were maintained in 48-well plates (Corning) with routine medium changes.

Effector function was quantified by IFN-γ ELISpot using a human IFN-γ ELISpot PRO (ALP) kit (Mabtech, Nacka Strand, Sweden; Cat. 3420-2AST-10/120), read on an iSpot reader (AID, Strassberg, Germany). CTL3 effectors (1 × 10^4^) were co-incubated with T2 cells (effector:T2 = 1:2) pulsed with the corresponding wild-type peptide, so every read-out measured native-epitope recognition. Each condition included a negative control (effectors plus unpulsed T2) and a positive control (effectors plus T2 plus phytohaemagglutinin [PHA; in-house preparation], 5 µg/mL); test and negative conditions were run in triplicate and positive controls in duplicate. Responses are expressed as spot-forming units per 10^6^ cells (SFU/10^6^) after subtraction of the matched negative control. A response was considered positive when the experimental/negative-control spot ratio was ≥1.4 and the well contained more than 15 spots. (Raw per-well spot counts in are reported separately as SFC/well.)

### 2.5. In Vitro Cytotoxicity Assay (CFSE/7-AAD) and HLA Class I Blockade

meHPV-CTLs (effectors; shown as HPV-T in the panels) were generated by stimulating CD8^+^ T cells from HLA-A*02:01-positive healthy donors with the six-peptide pool (E6wt, E7wt1, E7wt2, E6apl, E7apl2, and E7apl3), as described in Section 2.4. Mock-T cells (non-specific effector control) were generated from CD8^+^ T cells activated in vitro with anti-CD3 (clone OKT3, 30 ng/mL) and anti-CD28 (30 ng/mL) antibodies (R&D Systems, Minneapolis, MN, USA) under a rapid expansion protocol (REP) and expanded with the same interleukin cocktail used for HPV-T cells (IL-21, IL-7, IL-15, and IL-2, each at 10 ng/mL), without HPV16-peptide stimulation; they therefore lack HPV16-specific recognition of antigen-expressing target cells. Cytotoxicity was measured by a CFSE/7-AAD flow-cytometric live/dead assay using the standard reagents carboxyfluorescein succinimidyl ester (CFSE; Thermo Fisher Scientific, Waltham, MA, USA) and 7-aminoactinomycin D (7-AAD; BioLegend, San Diego, CA, USA). CFSE-labelled target cells (SiHa-0201-E7 or parental SiHa) were co-cultured with effectors at effector-to-target (E:T) ratios of 3:1, 10:1, and 30:1 for 21 h, and dead cells were identified by 7-AAD uptake on a CytoFLEX flow cytometer (Beckman Coulter, Brea, CA, USA) and analyzed with FlowJo software (BD Biosciences, Ashland, OR, USA). Specific lysis was calculated as: Specific lysis (%) = 100 × [(% dead_experimental_ − % dead_spontaneous_)/(100 − % dead_spontaneous_)], where spontaneous death was determined from target-only control wells. For HLA class I blockade, target cells were pre-incubated with the well-characterized pan-HLA class I monoclonal antibody clone W6/32 (mouse IgG2a) [27] (10 µg/mL; 37 °C; 30 min) before adding effectors, and cytotoxicity was evaluated at E:T = 30:1.

### 2.6. Molecular Dynamics Simulations and MM-GBSA Calculations

Molecular dynamics (MD) simulations were performed retrospectively, after the candidate peptides had been established experimentally, to interpret the observed differences in HLA-A*02:01 binding and immunogenicity at the structural level. Simulations covered the three simulated WT/APL pairs E6wt/E6apl, E7wt1/E7apl1, and E7wt2/E7apl2 (six peptides). This set deliberately differs from the six-peptide functional pool: E7apl2 was modelled as the representative of the two near-identical E7wt2-derived ligands (E7apl3 not simulated separately), In contrast, E7apl1 was included to provide a structural explanation for its high predicted binding but low effector immunogenicity. In addition, because the E7-1 position could not be tested in the T2 assay, MD simulations supplied vital structural evidence for the binding capacity of that pair.

Peptide–HLA-A*02:01 complexes were modelled on the crystallographic templates PDB 1DUZ (9-mer) [28] and PDB 1I4F (10-mer) [29], with substituted side chains built in PyMOL (Schrödinger, LLC, New York, NY, USA) [30]. Systems were simulated in AMBER 26 and AmberTools 26 with the ff19SB force field [31] and the OPC water model [32]; a single 50 ns production trajectory per complex was generated with the GPU-accelerated pmemd.cuda engine [33,34], with coordinates saved every 5 ps (10,000 frames over 50 ns).

Trajectories were analyzed with cpptraj [35,36]: each 50 ns production trajectory was autoimaged and stripped of water and ions (mask ‘:WAT, Na+, Cl-’). Backbone RMSD was calculated using HLA-A*02:01 Cα atoms (mask ‘:1-376’) referenced to the first frame; per-residue peptide RMSF was computed (byres) over the peptide mask (9-mer, ‘:376-384’; 10-mer, ‘:376-385’); and peptide-HLA hydrogen bonds were enumerated over the whole complex.

MM-GBSA binding free energies were computed with MMPBSA.py [35], using ante-MMPBSA.py for topology decomposition (receptor = HLA-A*02:01 plus β_2_-microglobulin, mask ‘:1-376’; ligand = peptide), with the GB model igb = 5, a salt concentration of 0.15 M, and internal/external dielectric constants left at their defaults (1.0/80.0). Energies were computed over the equilibrated 20–50 ns window, which contains 6000 trajectory frames (frames 4001–10,000 of the 10,000-frame trajectory, saved every 5 ps); MM-GBSA itself sampled every 10th frame (interval = 10), yielding ~600 frames per complex. Conformational entropy (−TΔS) was omitted, as normal mode analysis typically introduces large statistical uncertainties that degrade the accuracy of relative binding energy rankings for similar ligands. The resulting energies were interpreted comparatively among closely related ligands rather than as absolute binding free energies.

To further evaluate the temporal robustness of the structural and energetic analyses, block-averaged calculations were performed on the equilibrated 20–50 ns trajectories. Each trajectory was divided into three consecutive 10 ns blocks (20–30 ns, 30–40 ns, and 40–50 ns), and RMSF as well as MM-GBSA calculations were independently repeated for each block using identical parameters. Block-level values were summarized as mean ± standard error of the mean (SEM) to evaluate the consistency of the observed conformational and energetic trends across the equilibrated simulation period.

### 2.7. COCOMAPS 2.0-Based Peptide–HLA Interaction Analysis

Representative peptide–HLA-A*02:01 conformations extracted from equilibrated molecular dynamics trajectories were further analyzed using COCOMAPS 2.0 [37]. to characterize residue-level interaction patterns within the peptide–MHC interface.

The equilibrated structures were prepared in PDB format, with HLA-A*02:01 and peptide assigned as receptor and ligand components, respectively. COCOMAPS 2.0 analysis was performed to identify intermolecular contacts, including hydrogen bonds, CH–O/N bonds, polar and apolar van der Waals interactions, π-related interactions, and proximal contacts.

Interaction parameters, including buried interface area, residue-level contact contributions, and interaction hotspot profiles, were extracted and compared between WT and corresponding APL complexes. Differential interaction patterns were calculated relative to the corresponding WT complexes and used for visualization in Supplementary Figure S3.

### 2.8. HPV16 Sequence Conservation Analysis

HPV16 E6 and E7 sequence conservation analysis was performed using representative HPV16 genomic sequences retrieved from the NCBI GenBank database [19]. Sixteen representative HPV16 variants covering major phylogenetic lineages (A1–A4, B1–B4, C1–C4, and D1–D4) were included.

Coding sequences of E6 and E7 were extracted and translated into amino acid sequences using a customized Python workflow based on Biopython [38]. Multiple sequence alignment was performed using MAFFT version 7.453 with the L-INS-i algorithm (--localpair --maxiterate 1000) [39].

Residue-level conservation was calculated based on the frequency of the dominant amino acid at each alignment position after excluding gap positions. Conservation profiles were generated for full-length HPV16 E6/E7 proteins and selected epitopes.

### 2.9. HLA-A*02:01 Population Coverage Analysis

Population coverage analysis was performed using the IEDB population coverage tool [21], based on the algorithm developed by Bui et al. [40], to estimate the potential applicability of the selected HPV16 epitopes in HLA-A*02:01-positive populations.

The final six-peptide formulation (E6wt, E7wt1, E7wt2, E6apl, E7apl2, and E7apl3) was used as input, with HLA-A*02:01 specified as the restricting allele. Coverage estimates were calculated for worldwide, East Asian, European, and North American reference populations provided by the IEDB database.

### 2.10. Statistical Analysis

**Figure 1:**
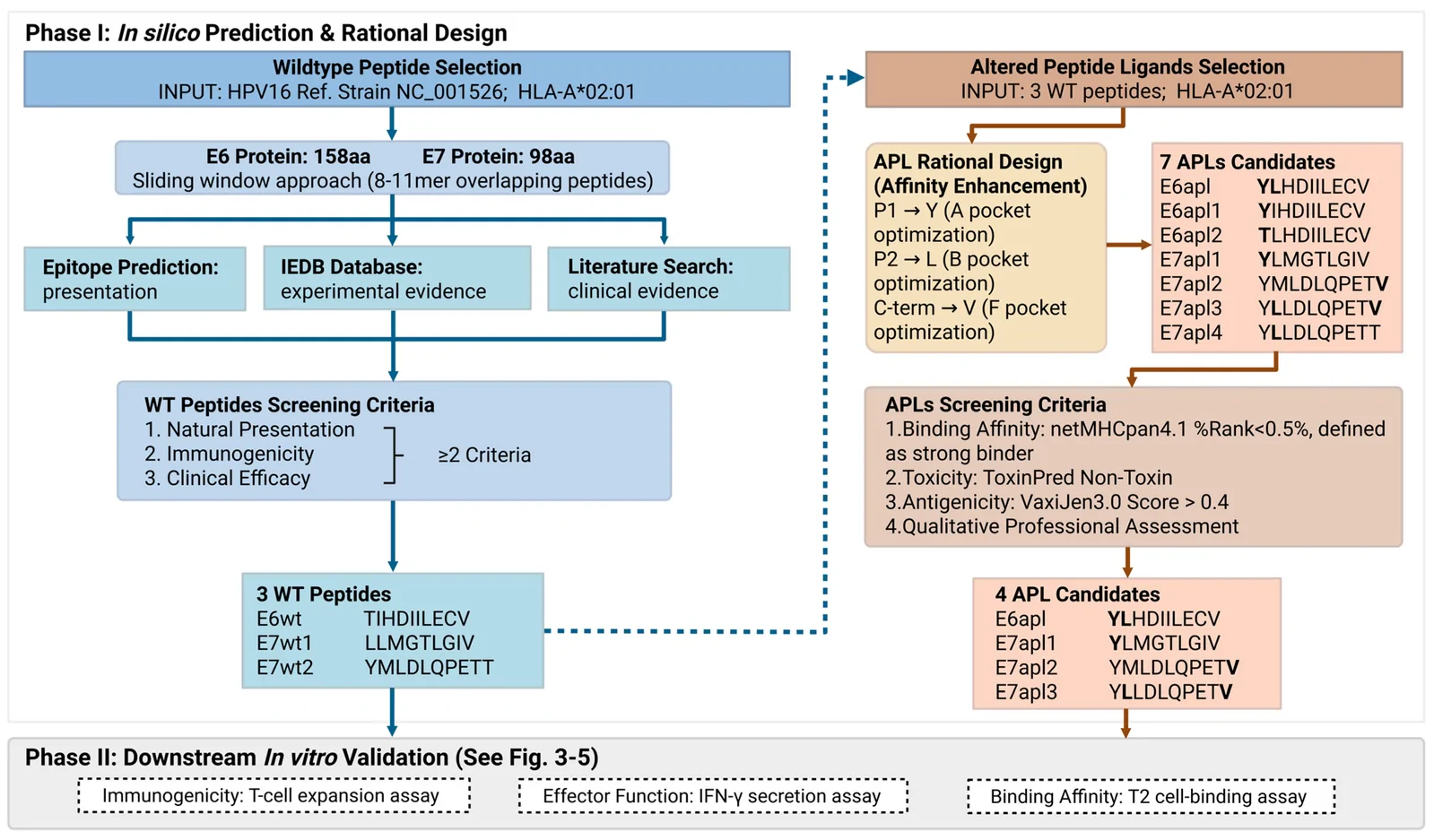
Immunoinformatics-guided rational design and experimental validation of multi-anchor altered peptide ligands (APLs) targeting HPV16 E6 and E7 oncoproteins. The workflow consists of two sequential phases. Phase I (In silico prediction and rational design): Candidate HLA-A*02:01-restricted epitopes were derived from HPV16 E6 (158 aa) and E7 (98 aa) reference sequences (NC_001526) using an 8–11mer sliding-window approach. Epitope prioritization integrated NetMHCpan-4.1 binding predictions, IEDB-curated experimental evidence, and literature-derived clinical annotations. Three wild-type (WT) epitopes were selected based on these criteria: E6wt (TIHDIILECV), E7wt1 (LLMGTLGIV), and E7wt2 (YMLDLQPETT). APLs were generated using a multi-anchor optimization strategy targeting HLA-A*02:01 anchor positions (P1 → Y, A-pocket; P2 → L, B-pocket; C-terminal → V, F-pocket). Candidate peptides were filtered based on predicted binding affinity (%Rank < 0.5), predicted non-toxicity (ToxinPred), and antigenicity (VaxiJen > 0.4), followed by expert curation, yielding four prioritized APLs (E6apl, E7apl1, E7apl2, and E7apl3). Phase II (Experimental validation): Selected WT and APL peptides were evaluated in vitro for HLA-A*02:01 binding (T2 stabilization assay), T-cell expansion, and effector function (IFN-γ ELISpot), as detailed in Figures 3–5. Peptide nomenclature follows Table 1.

**Figure 2:**
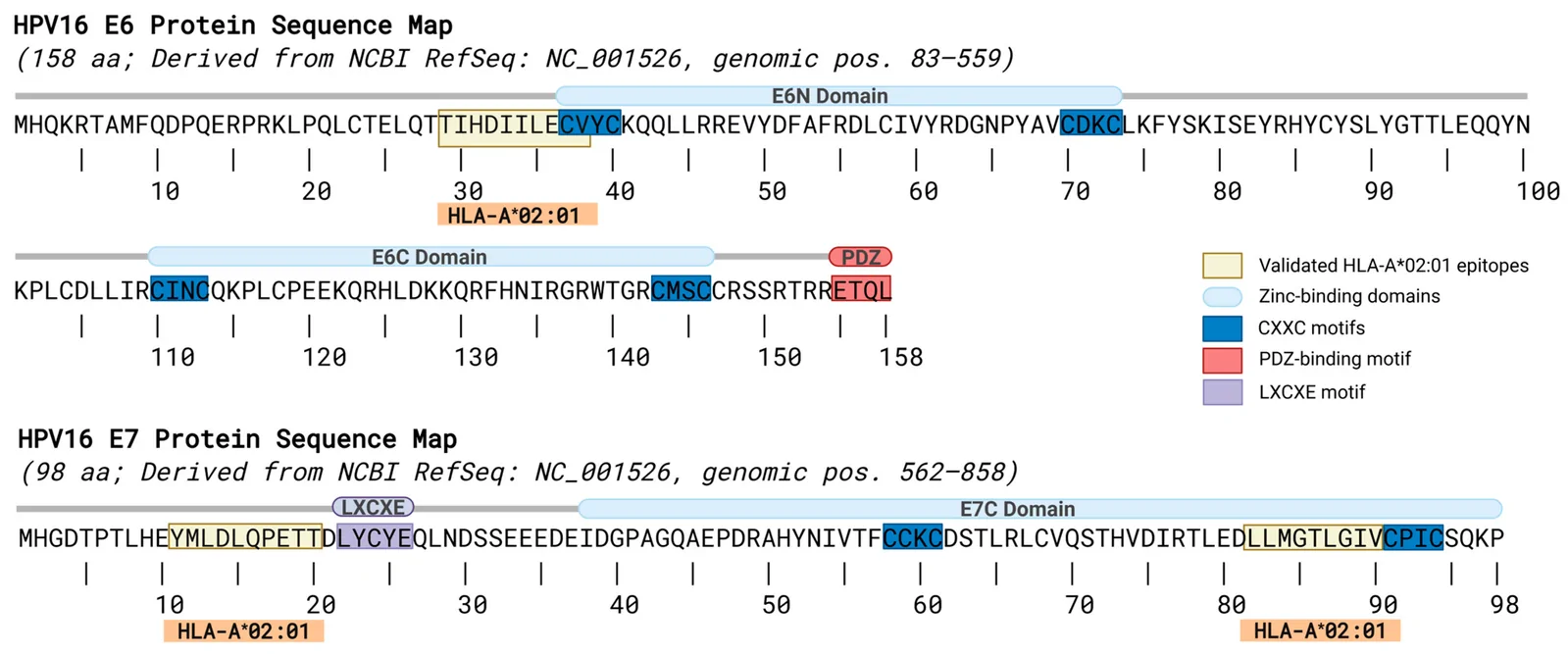
Linear sequence mapping of HLA-A*02:01-restricted wild-type epitopes onto the functional domain architecture of HPV16 E6 and E7 oncoproteins. Full-length amino acid sequences with annotated functional domains are depicted for the HPV16 E6 protein (158 aa; genomic positions 83–559) and the HPV16 E7 protein (98 aa; genomic positions 562–858), based on the NCBI reference sequence NC_001526. The annotated schematics delineate key structural and oncogenic features, including the N-terminal (E6N) and C-terminal (E6C) domains of E6, conserved zinc-binding domains defined by canonical Cys–x–x–Cys (CXXC) motifs, the C-terminal PDZ-binding motif of E6, and the LXCXE motif of E7 that mediates retinoblastoma protein (pRb) binding and functional inactivation. The sequence positions of the three prioritized wild-type epitopes—E6wt (TIHDIILECV), E7wt1 (LLMGTLGIV), and E7wt2 (YMLDLQPETT)—are annotated directly on the respective protein maps, illustrating their localization relative to key oncogenic functional domains.

Schematic illustrations (Figures 1 and 2) were created using BioRender.com. Statistical analyses and data visualization were performed in R software v4.4.3 (R Foundation for Statistical Computing, Vienna, Austria), using ggplot2 (v4.0.3) and dplyr (v1.2.1).

For cytotoxicity assays, technical replicates were averaged for each donor prior to statistical analysis. Biological replicates consisted of independent healthy donors (*n* = 3, HD1–HD3), with Mock-T serving as a shared internal control. Data are presented as mean ± standard error of the mean (SEM). Statistical significance was assessed using a two-sided one-sample Student’s *t*-test against Mock-T, a two-sided paired Student’s *t*-test for target specificity (SiHa-0201-E7 vs. parental SiHa, and a two-sided paired Student’s *t*-test for HLA class I blockade experiments (±W6/32 antibody).

**Figure 3:**
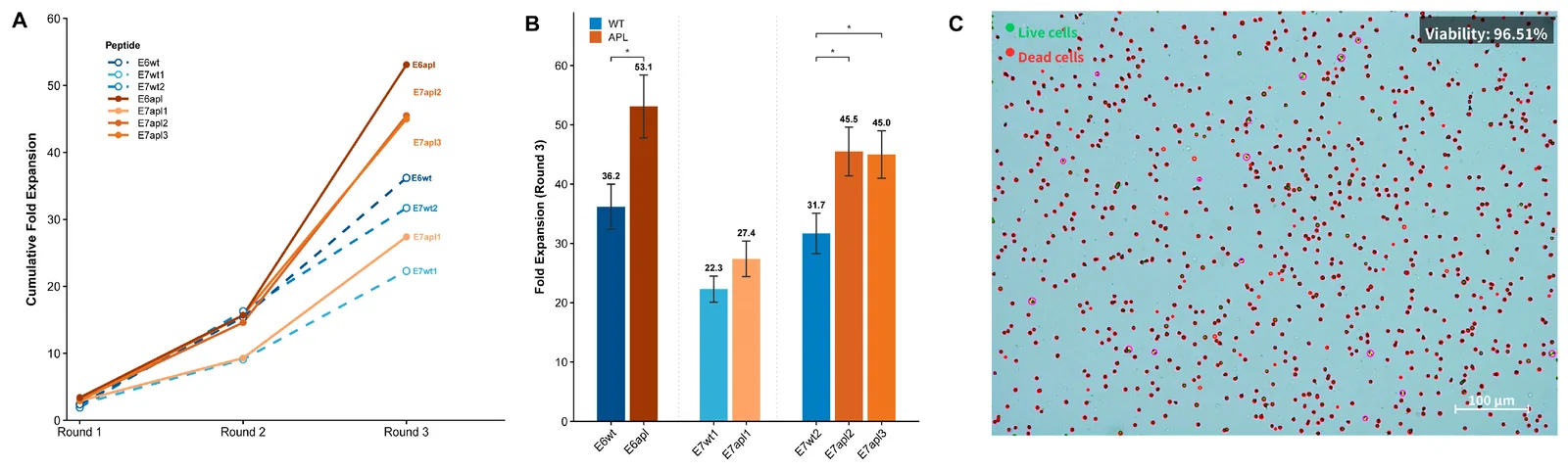
HPV16 E6- and E7-derived altered peptide ligands (APLs) enhanced CD8^+^ T-cell expansion during repeated peptide stimulation. (**A**) Cumulative fold expansion of CD8^+^ T cells across three rounds of stimulation. HLA-A*02:01^+^ donor-derived CD8^+^ T cells were stimulated with autologous peptide-pulsed dendritic cells over three sequential rounds (7–9 days each). Wild-type (WT) peptides are shown as dashed lines with open circles, and APLs are shown as solid lines with filled circles. Colors indicate individual peptides as shown in the legend. Data are shown for a representative HLA-A*02:01^+^ donor. (**B**) Total fold expansion after three stimulation rounds. Bars represent mean ± SD from three replicate expansion measurements (*n* = 3). Peptides are grouped by antigen lineage and corresponding WT sequence (E6wt/E6apl; E7wt1/E7apl1; E7wt2/E7apl2/E7apl3). Statistical comparisons were performed using two-tailed unpaired t-tests (* *p* < 0.05); only statistically significant differences are shown. (**C**) Representative acridine orange/propidium iodide (AO/PI) fluorescence image of E7apl2-expanded CD8^+^ T cells on day 26 after stimulation. Viable cells exhibit green fluorescence, whereas dead cells exhibit red fluorescence. Red circular marks represent automated cell-identification overlays generated during image analysis and do not indicate dead-cell staining. Cell viability was 96.51% at a density of 3.8 × 10^6^ cells/mL. Scale bar: 100 μm.

**Figure 4:**
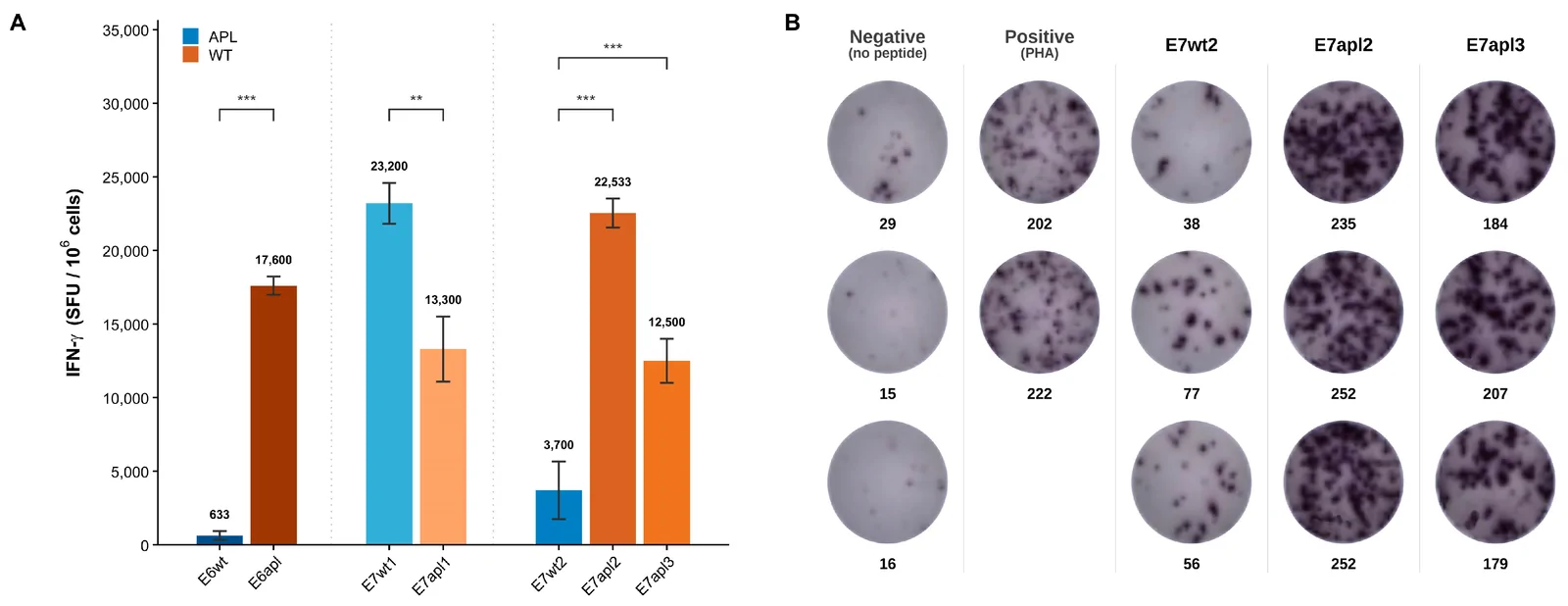
IFN-γ ELISpot analysis of CD8^+^ CTL responses induced by HPV16 E6/E7-derived wild-type peptides and altered peptide ligands (APLs). (**A**) Quantification of IFN-γ ELISpot responses following three rounds of peptide stimulation. Data are presented as mean ± SD (*n* = 3 replicate ELISpot measurements) and expressed as spot-forming units (SFU) per 10^6^ cells after subtraction of background signals from unpulsed controls. Statistical comparisons between WT peptides and corresponding APLs were performed using one-way ANOVA followed by Dunnett’s multiple comparisons test. (** *p* < 0.01, *** *p* < 0.001). (**B**) Representative ELISpot wells showing IFN-γ responses of CTLs generated with the HPV16 E7_11−20_ wild-type peptide (E7wt2; YMLDLQPETT) or its corresponding APLs (E7apl2: YMLDLQPETV; E7apl3: YLLDLQPETV). To evaluate recognition of the native epitope, all expanded CTLs were evaluated using T2 target cells pulsed with the corresponding wild-type peptide (effector: T2 = 1:2). Negative controls consisted of CTLs incubated with unpulsed T2 cells, and positive controls consisted of CTLs stimulated with PHA (5 µg/mL). Raw spot counts (SFC/well) are indicated beneath each well. Data are representative of four independent CTL induction experiments performed using cells from the same donor.

For in vitro T-cell expansion (Figure 3) and ELISpot assays (Figure 4), experiments were performed using technical replicates across three peptide-stimulated cultures. Due to limited donor stratification in these exploratory assays, statistical comparisons were performed at the experimental-replicate level and are presented as descriptive quantitative comparisons. Data are shown as mean ± standard deviation (SD). Expansion data (Figure 3B) were analyzed using two-sided unpaired Student’s *t*-tests. ELISpot responses (Figure 4) were analyzed using one-way ANOVA followed by Dunnett’s multiple comparisons test, with each APL compared with its corresponding WT peptide. Adjusted *p* values were calculated using the single-step method.

Statistical significance was defined as *p* < 0.05, * *p* < 0.01, and ** *p* < 0.001 for individual comparisons. For multiple-comparison analyses (Figure 4), adjusted *p* values were calculated using Dunnett’s multiple comparisons test, and statistical significance was defined as adjusted *p* < 0.05.

## 3. Results

### 3.1. Immunoinformatics-Based Identification and Prioritization of HPV16 E6/E7 Epitopes and APL Candidates

We utilized an immunoinformatics-driven screening pipeline to identify and prioritize HLA-A*02:01-restricted HPV16 E6 and E7 epitopes with improved predicted presentation (Figure 1). The integration of NetMHCpan-4.1 predictions, IEDB-derived epitope annotations, and literature-reported clinical evidence converged on three lead wild-type (WT) epitopes: E6wt (TIHDIILECV), E7wt1 (LLMGTLGIV), and E7wt2 (YMLDLQPETT).

Anchor-residue optimization at canonical HLA-A*02:01 positions (P1 → Y, P2 → L, and the C-terminal residue → V) generated seven candidate APLs. After sequential computational filtering based on binding affinity (%Rank < 0.5%), predicted non-toxicity, and antigenicity, four computationally prioritized APLs (E6apl, E7apl1, E7apl2, and E7apl3) were selected for downstream analyses (Table 1).

Although absolute predicted affinities varied across peptides (12.59–329.33 nM), all WT epitopes were classified as weak HLA-A*02:01 binders based on %Rank values above the 0.5% threshold (0.990–1.442%). In contrast, all APL variants exhibited markedly improved predicted binding, with %Rank values below 0.5% (0.024–0.298%) and corresponding affinities in the low nanomolar range (4.24–6.63 nM).

Consistently, predicted immunogenicity scores shifted from negative values for WT epitopes to positive values for all APLs (0.111–0.335), while whole-peptide antigenicity and toxicity profiles remained uniformly favorable within each epitope lineage. Of note, among the four APLs, E7apl1 showed the relatively weakest predicted presentation score despite robust binding affinity.

Mutated residues in APL sequences are highlighted in bold. APL, altered peptide ligand; WT, wild-type; SB, strong binder; WB, weak binder. Immunogenicity scores were predicted using the IEDB Class I Immunogenicity Predictor; antigenicity using VaxiJen v3.0; and toxicity using ToxinPred. Presentation scores, %Rank values, binding affinities, and binding classifications were predicted using NetMHCpan-4.1 (%Rank < 0.5% = strong binder, SB; 0.5–2.0% = weak binder, WB).

Structural mapping of the selected epitopes (E6wt, residues 29–38; E7wt2, residues 11–20; E7wt1, residues 82–90) onto the HPV16 E6 and E7 domain architecture (Figure 2) revealed localization within structurally constrained regions. The mutations of these epitopes do not overlap with the zinc-binding domain in E6 or the pRb-binding LXCXE motif in E7, thus minimizing the risk of restoring oncogenic functions; detailed motif positions are annotated in Figure 2.

To further evaluate the evolutionary conservation of the selected HPV16 targets, the amino acid conservation of the E6 and E7 proteins was analyzed across 16 representative HPV16 sublineages. The regions encompassing the selected WT epitopes and corresponding APL optimization sites showed high conservation across the analyzed sequences (Figure S1). Furthermore, residue-level analysis of the WT epitopes and APL substitution positions demonstrated that all introduced modification sites were located at highly conserved viral residues, with 100% conservation of the targeted natural residues (Figure S2 and Table S1). These findings indicate that the designed APL substitutions were introduced at conserved HPV16 positions rather than naturally variable regions.

The overall experimental workflow, including computational prediction, HLA-A*02:01 stabilization assessment, T-cell functional evaluation, structural analysis, and final formulation selection for each peptide candidate, is summarized in Table S2.

### 3.2. APL Stimulation Enhances CD8^+^ T-Cell Expansion Profiles

To assess whether anchor optimization improved CD8^+^ T-cell proliferation, HLA-A*02:01^+^ donor-derived CD8^+^ T cells were stimulated with autologous peptide-pulsed dendritic cells over three sequential rounds (7–9 days each; Figure 3).

Cumulative fold expansion consistently increased across the three stimulation rounds (Figure 3A). By the end of the third round, APL-treated cultures showed increased CD8^+^ T-cell expansion compared with their corresponding WT peptide controls in replicate expansion measurements (Figure 3B; mean ± SD from three replicate expansion measurements, *n* = 3). Specifically, E6apl induced a 53.1 ± 5.3-fold expansion versus 36.2 ± 3.8-fold for E6wt (*p* = 0.011). Similarly, E7apl2 (45.5 ± 4.1-fold) and E7apl3 (45.0 ± 4.0-fold) showed higher expansion compared with E7wt2 (31.7 ± 3.4-fold; *p* = 0.011 and *p* = 0.012, respectively). E7apl1 followed a similar trend but did not reach statistical significance (27.4 ± 3.0-fold versus 22.3 ± 2.3-fold for E7wt1; *p* = 0.078).

Throughout the culture period, expanded populations maintained high viability. A representative E7apl2-stimulated culture at day 26 showed 96.51% viability at a density of 3.8 × 10^6^ cells/mL, as assessed by AO/PI staining (Figure 3C).

### 3.3. ELISpot Analysis Reveals Divergent Effector Responses and E7apl1-Specific Functional Discordance

To evaluate antigen-specific effector responses after peptide stimulation, IFN-γ secretion by CD8^+^ T cells following three rounds of stimulation was assessed using T2 cells pulsed with the corresponding wild-type (WT) epitopes (Figure 4A). Antigen-specific spot-forming units (SFUs) were calculated after subtraction of background responses from unpulsed control wells. Statistical comparisons were performed using one-way ANOVA followed by Dunnett’s multiple comparisons test. Among the evaluated epitope pairs, APL stimulation resulted in increased IFN-γ responses for two peptide lineages compared with their corresponding WT controls. Specifically, E6apl elicited 17,600 ± 625 SFU/10^6^ cells compared with 633 ± 289 SFU/10^6^ cells for E6wt (*p* = 1.80 × 10^−6^). Similarly, E7apl2 (22,533 ± 981 SFU/10^6^ cells; *p* = 9.71 × 10^−6^) and E7apl3 (12,500 ± 1493 SFU/10^6^ cells; *p* = 7.35 × 10^−4^) showed higher responses than E7wt2 (3700 ± 1952 SFU/10^6^ cells).

In contrast, the E7wt1/E7apl1 pair exhibited an inverse pattern, with E7wt1 inducing higher IFN-γ responses than E7apl1 (23,200 ± 1389 versus 13,300 ± 2207 SFU/10^6^ cells; *p* = 0.0028). This result indicates that improved predicted peptide–HLA-A*02:01 binding does not necessarily correspond to enhanced effector T-cell responses, highlighting the importance of functional evaluation during APL selection. Consequently, E7apl1 was excluded from the final multi-epitope formulation used in subsequent cytotoxicity assays.

### 3.4. APLs Exhibit Enhanced HLA-A*02:01 Stabilization In Vitro

**Figure 5:**
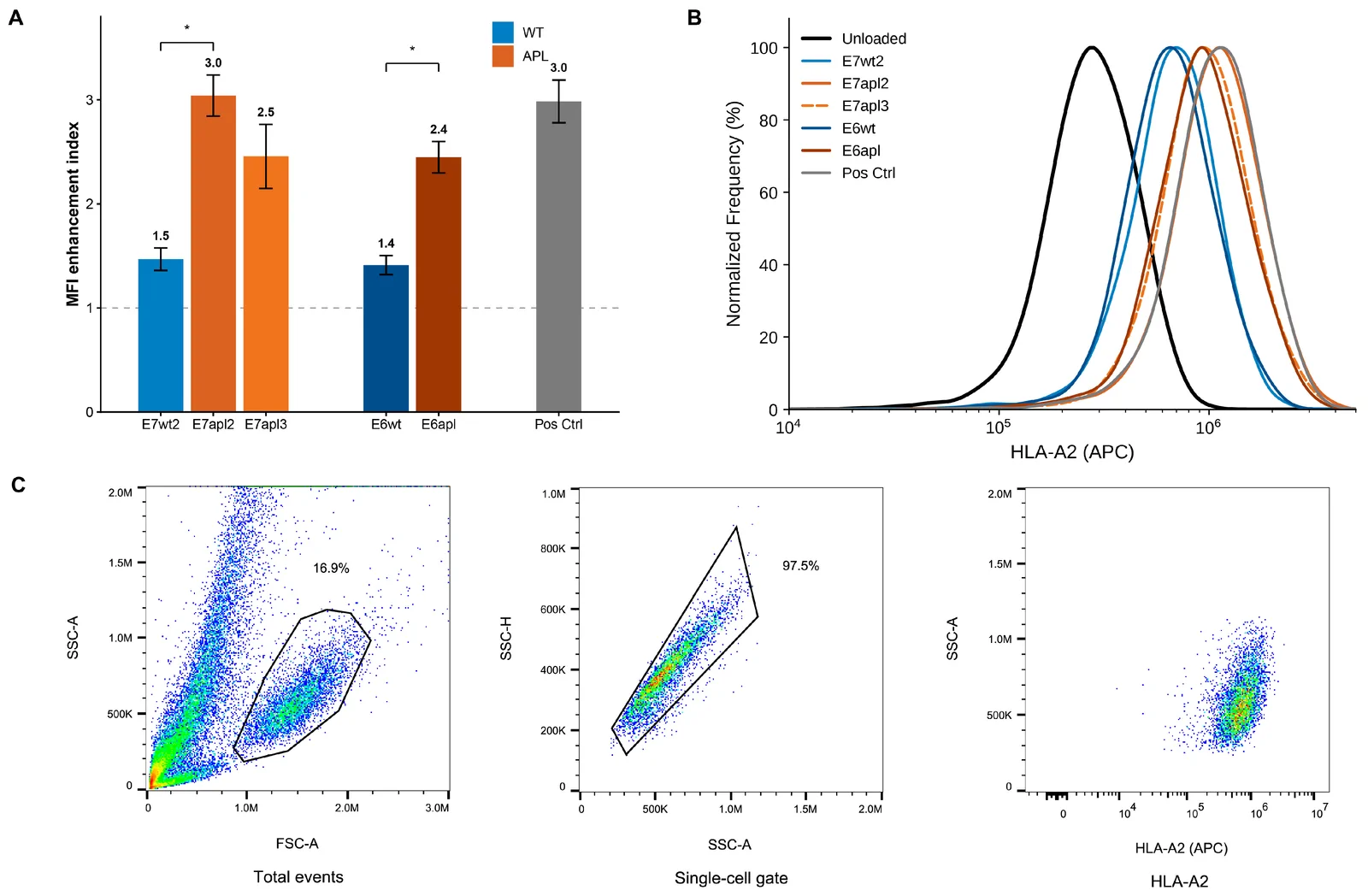
HLA-A*02:01 stabilization capacity of HPV16 E6/E7 wild-type (WT) peptides and altered peptide ligands (APLs) assessed by the T2-cell stabilization assay. TAP-deficient HLA-A*02:01^+^ T2 cells were incubated with the indicated peptides (100 µg/mL, overnight), and surface HLA-A*02:01 stabilization was quantified by flow cytometry using an APC-conjugated anti-HLA-A2 antibody. Data are presented as mean ± SD from duplicate measurements (n = 2). (**A**) MFI enhancement index, calculated as (MFI_peptide_ − MFI_unloaded_)/MFI_unloaded_. The dashed line indicates the threshold for peptide-induced stabilization (index ≥ 1). WT peptides are shown in blue and APLs in orange. E6apl, E7apl2, and E7apl3 displayed increased HLA-A*02:01 stabilization compared with their cognate WT peptides. Statistical comparisons between WT peptides and corresponding APLs were performed using two-tailed unpaired Welch’s *t*-tests (* *p* < 0.05). (**B**) Representative overlaid histograms of surface HLA-A2 (APC) staining for unloaded (no peptide) and peptide-pulsed T2 cells. Histograms are normalized to mode; rightward shifts indicate increased peptide-dependent stabilization of surface HLA-A*02:01 relative to unloaded controls. (**C**) Representative gating strategy for T2 flow cytometry analysis. Cells were first selected based on FSC-A and SSC-A characteristics, followed by singlet discrimination using SSC-A versus SSC-H. Surface HLA-A2 expression was subsequently analyzed within the gated single-cell population. A TWISTNB-derived HLA-A*02:01-restricted peptide (KLMGIVYKV) served as the positive control (Pos Ctrl).

To experimentally evaluate peptide-dependent HLA-A02:01 stabilization, TAP-deficient HLA-A*02:01 T2 cells were incubated with the indicated peptides, and surface HLA-A*02:01 expression was quantified by flow cytometry using the MFI enhancement index (Figure 5; mean of *n* = 2 duplicate measurements). The MFI enhancement index was calculated as (MFI_peptide_ − MFI_unloaded_)/MFI_unloaded_. All evaluated peptides exceeded the predefined stabilization threshold (index ≥ 1; Figure 5A).WT epitopes showed moderate HLA-A*02:01 stabilization, with E7wt2 and E6wt exhibiting MFI enhancement indices of 1.47 and 1.41, respectively. In comparison, the corresponding APLs demonstrated increased stabilization capacity, with E7apl2, E7apl3, and E6apl showing indices of 3.04, 2.46, and 2.45. Statistical comparisons between WT peptides and their corresponding APLs indicated significantly increased stabilization for E7apl2 versus E7wt2, E7apl3 versus E7wt2, and E6apl versus E6wt (Figure 5A). The stabilization index of E7apl2 was comparable to that of the reference positive-control peptide KLMGIVYKV (3.04 versus 2.98). Representative flow cytometry histograms and the gating strategy are shown in Figure 5B,C.

The E7wt1/E7apl1 pair was not evaluated in the T2 stabilization assay because of peptide solubility limitations under the non-toxic DMSO conditions used in this study. Therefore, the predicted HLA-A*02:01 presentation characteristics of this epitope pair were considered based on computational prediction (Table 1), while structural analyses were further performed to explore potential peptide–HLA interaction properties.

### 3.5. meHPV-CTLs Mediate Potent, Target-Specific, and HLA Class I-Restricted Cytotoxicity

meHPV-CTLs (designated as HPV-T in the figures) were generated using the six-peptide pool consisting of E6wt, E7wt1, E7wt2, E6apl, E7apl2, and E7apl3, and were subsequently evaluated against the HLA-matched, E7-expressing target cells SiHa-0201-E7. The final peptide pool excluded E7apl1 because this APL showed reduced IFN-γ effector responses compared with its corresponding WT peptide (Figure 4).

**Figure 6:**
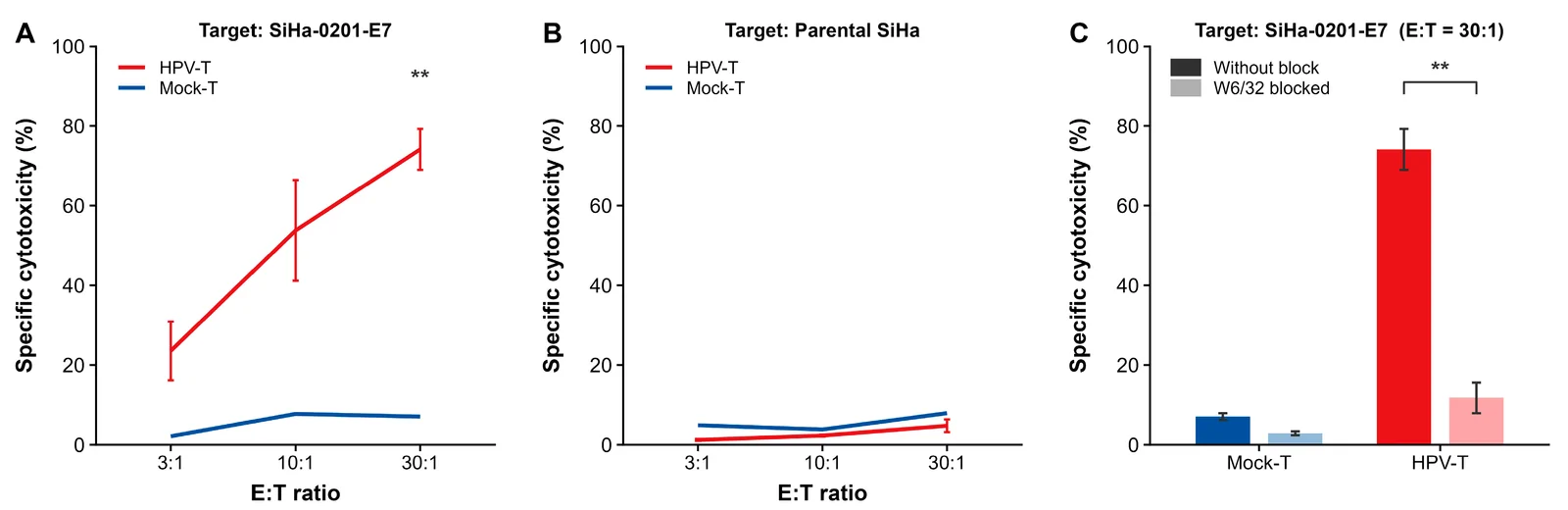
Cytotoxic activity of meHPV-CTLs (HPV-T) against cervical carcinoma targets assessed by CFSE/7-AAD assay (21 h). Data for meHPV-CTLs (HPV-T) are presented as mean ± SEM from three healthy donors (HD1–HD3); technical duplicates were averaged per donor. Mock-T cells served as a shared effector control without donor-level tracking. (**A**) Dose-dependent specific lysis of HLA-matched, E7-expressing SiHa-0201-E7 cells by meHPV-CTLs compared with Mock-T cells at E:T ratios of 3:1, 10:1, and 30:1. Specific lysis by meHPV-CTLs increased to 23.5 ± 7.4%, 53.8 ± 12.6%, and 74.1 ± 5.1%, respectively, whereas Mock-T controls showed background-level lysis (≤8%). Statistical significance was assessed using a two-sided one-sample *t*-test comparing HPV-T responses with Mock-T control values (*p* < 0.01 at 30:1). (**B**) Target specificity was assessed using parental SiHa cells. At an E:T ratio of 30:1, meHPV-CTLs induced minimal lysis of parental SiHa cells compared with SiHa-0201-E7 targets (4.7 ± 1.6% versus 74.1 ± 5.1%; *p* = 0.003). (**C**) HLA class I restriction was assessed at an E:T ratio of 30:1. Pre-incubation of SiHa-0201-E7 cells with the pan-HLA class I antibody W6/32 markedly reduced specific lysis from 74.1 ± 5.1% to 11.7 ± 3.9% (paired *t-*test, *p* = 0.006), supporting HLA class I-dependent target recognition. (** *p* < 0.01).

The meHPV-CTLs exhibited dose-dependent cytotoxicity against SiHa-0201-E7 cells, with specific lysis increasing across the tested effector-to-target (E:T) ratios (3:1, 23.5 ± 7.4%; 10:1, 53.8 ± 12.6%; and 30:1, 74.1 ± 5.1%). These values represent mean ± SEM from three healthy donors (HD1–HD3). In contrast, Mock-T cells, used as a shared effector control without donor-level tracking, showed only background-level lysis (≤8%) across the tested conditions. At the highest E:T ratio (30:1), meHPV-CTL-mediated cytotoxicity was significantly higher than that observed for Mock-T controls (*p* < 0.01) (Figure 6A).

To further evaluate target specificity, meHPV-CTLs were tested against parental SiHa cells lacking the introduced HLA-A*02:01-restricted E7 antigen presentation system. At an E:T ratio of 30:1, meHPV-CTLs induced minimal lysis of parental SiHa cells compared with SiHa-0201-E7 targets (4.7 ± 1.6% versus 74.1 ± 5.1%; *p* = 0.003), indicating preferential recognition of antigen-presenting target cells (Figure 6B).

The contribution of HLA class I molecules to target recognition was assessed using W6/32 antibody blockade. Pre-incubation of SiHa-0201-E7 cells with W6/32 markedly reduced specific lysis from 74.1 ± 5.1% to 11.7 ± 3.9% at an E:T ratio of 30:1 (~80% reduction; *p* < 0.01), supporting the HLA class I dependence of meHPV-CTL-mediated cytotoxicity (Figure 6C).

### 3.6. Molecular Dynamics Reveals Structural Features Associated with pMHC Stability and the Binding–Immunogenicity Dissociation

To investigate the structural effects of the anchor-residue substitutions, 50 ns all-atom molecular dynamics (MD) simulations were performed for three WT/APL pairs (E6wt/E6apl, E7wt1/E7apl1, and E7wt2/E7apl2) in complex with HLA-A*02:01. E7apl2 was modeled as the representative for the E7wt2 lineage. In particular, E7apl1 was included because it exhibited a functional discordance characterized by favorable predicted HLA-A02:01 presentation but reduced effector responses, and its HLA stabilization could not be experimentally evaluated due to peptide solubility limitations.

**Figure 7:**
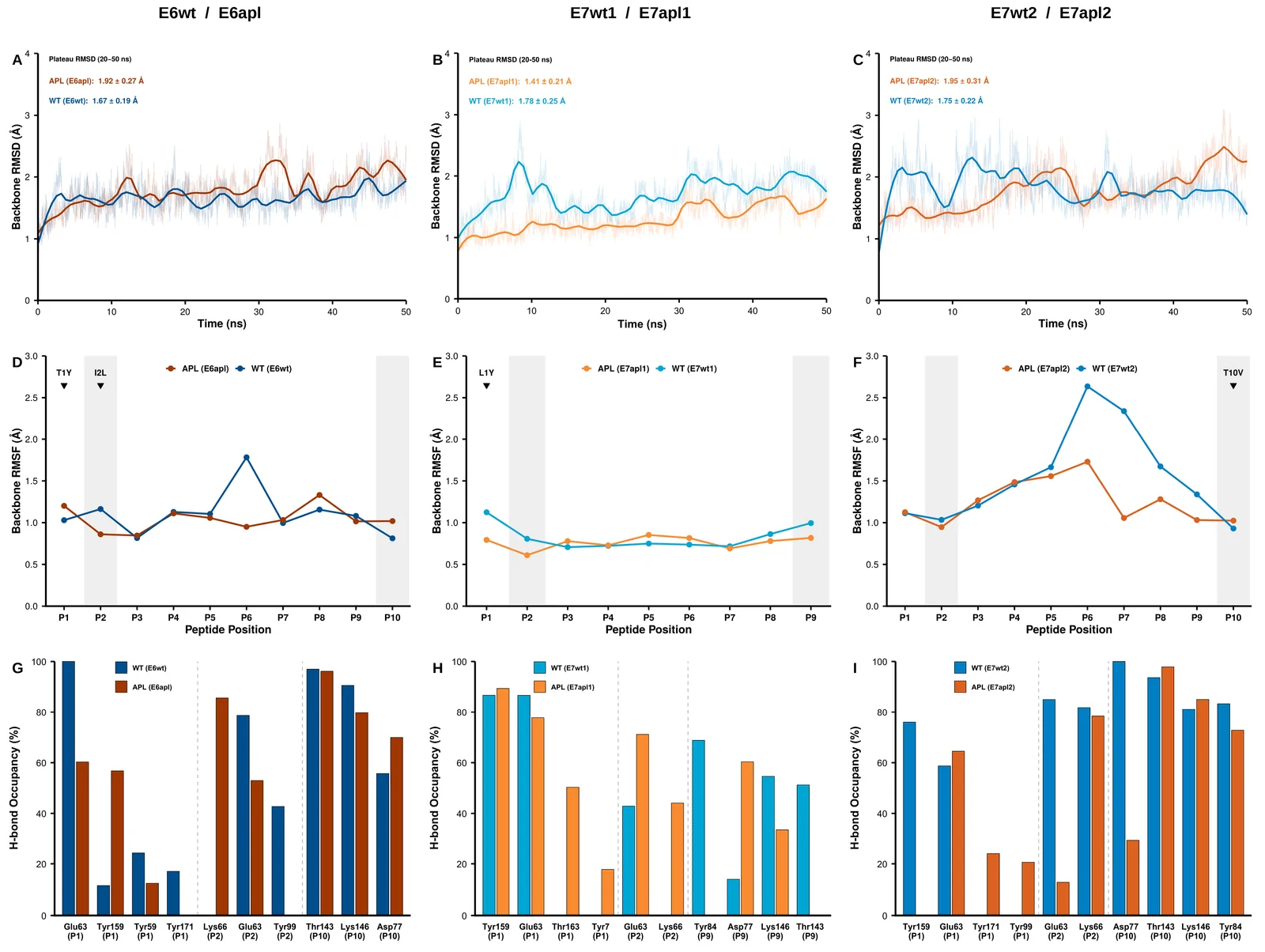
Molecular dynamics analysis of the three simulated WT/APL peptide–HLA-A*02:01 pairs (E6wt/E6apl, E7wt1/E7apl1, E7wt2/E7apl2). (**A**–**C**) Backbone RMSD over the 50 ns trajectories: all six complexes reached stable RMSD profiles within ~20 ns and fluctuate within ~1.4–2.0 Å over 20–50 ns. (**D**–**F**) Per-residue RMSF: the T10V substitution was associated with reduced flexibility in the central region of E7apl2 (P6 peak ~2.6 Å → ~1.0 Å); E6apl is similarly damped vs. E6wt; E7apl1 displayed reduced RMSF values compared with E7wt1 across multiple peptide positions, including central residues of the peptide. (**G**–**I**) Peptide–HLA hydrogen-bond occupancy: anchor pockets engage Glu63/Lys66 (P2) and Asp77/Thr143/Lys146 (C-terminus); the E7apl2 T10V substitution reduced the Thr–Asp77 hydrogen-bond occupancy (~100% to ~30%) while retaining other C-terminal contacts; E7apl1 L1→Y introduces additional P1 contacts. These structural observations are considered together with the MM-GBSA binding free energy profiles reported in Table 2.

Backbone RMSD profiles showed that all six complexes reached a stable plateau within the first ~20 ns and fluctuated within a relatively narrow range of approximately 1.4–2.0 Å over the 20–50 ns analysis window (Figure 7A–C), indicating that the peptide–HLA complexes remained structurally stable during the simulation period. Rather than displaying uniformly lower RMSD values, the APLs adopted equilibrium conformations that differed modestly from their WT counterparts, reflecting local rearrangements introduced by the substituted residues. Consistently, MM-GBSA calculations indicated favorable binding free energies (ΔG_bind_) across all complexes (Table 2). The calculated ΔG_bind_ values were comparable between the highly functional E7apl2 (−82.23 kcal/mol) and the functionally weaker E7apl1 (−80.21 kcal/mol), suggesting that overall thermodynamic stability alone may not fully explain the differences in functional T-cell responses.Within the E6 lineage, a similar pattern was observed. E6apl exhibited the weakest computed binding free energy among the simulated complexes (ΔG_bind_ = −73.59 kcal/mol; Table 2), yet displayed strong functional immunogenicity in vitro. Conversely, its parental counterpart E6wt showed a more favorable calculated binding energy (ΔG_bind_ = −84.14 kcal/mol). Together with the E7apl1/E7apl2 comparison, these observations indicate that calculated binding energetics do not strictly correspond to functional CTL responses among the evaluated peptide pairs.

The most pronounced effects of the substitutions were observed in the per-residue RMSF analysis (Figure 7D–F). For the E7wt2/E7apl2 pair, the C-terminal T10V substitution reduced the fluctuation of the central peptide region, particularly the prominent fluctuation peak around P6 observed in E7wt2 (~2.6 Å), which decreased to approximately ~1.0 Å in E7apl2. A similar conformational ordering was observed for E6apl relative to E6wt, where introduction of the optimized P2 (Leu) anchor was associated with reduced peptide flexibility. In contrast, E7apl1 (L1Y) displayed lower RMSF values across multiple peptide positions, including solvent-exposed regions, compared with E7wt1, indicating a more constrained peptide conformational profile within the HLA-A*02:01 groove.

Hydrogen-bond occupancy analysis further characterized the interaction patterns between peptides and HLA-A*02:01 (Figure 7G–I). Across all complexes, conserved anchor-region interactions involving Glu63 and Lys66 at the P2 region, as well as Asp77, Thr143, and Lys146 at the C-terminal region (PΩ), were maintained. The clearest substitution-associated change was observed in E7apl2, where the T10V replacement reduced the threonine-mediated hydrogen bond interaction with Asp77 (from approximately 100% occupancy in E7wt2 to approximately 30% in E7apl2). However, other C-terminal anchoring interactions remained preserved, suggesting a rearrangement of local interaction patterns following substitution. In addition, E7apl2 showed a strengthened electrostatic contribution (ΔE_elec_ = −299.54 kcal/mol; Table 2), consistent with its favorable functional performance.

To further evaluate the reproducibility of the observed structural trends, block-averaged analyses were performed using three consecutive 10 ns segments extracted from the equilibrated 20–50 ns trajectories (20–30 ns, 30–40 ns, and 40–50 ns; Figure S4). The RMSF profiles obtained from individual trajectory blocks showed similar residue-level fluctuation patterns for the E6wt/E6apl, E7wt1/E7apl1, and E7wt2/E7apl2 peptide–HLA complexes (Figure S4A–C). Likewise, block-wise MM-GBSA calculations showed comparable favorable binding energy profiles across the three trajectory segments, although moderate variations between individual blocks were observed (Figure S4D–F). These results support the stability of the major structural features identified from the original trajectories.

Taken together, these simulations provide structural insight into the relationship between peptide conformational properties and functional responses. In particular, E7apl1 exhibited reduced peptide flexibility compared with E7wt1 despite maintaining favorable predicted HLA-A*02:01 presentation characteristics. These findings suggest that altered conformational dynamics may contribute to the functional divergence observed for E7apl1, although direct pMHC–TCR structural or biophysical analyses will be required to establish a causal relationship between peptide flexibility and T-cell recognition.

### 3.7. COCOMAPS 2.0 Analysis Reveals Residue-Level Remodeling of Peptide–HLA-A*02:01 Interaction Networks

**Figure 8:**
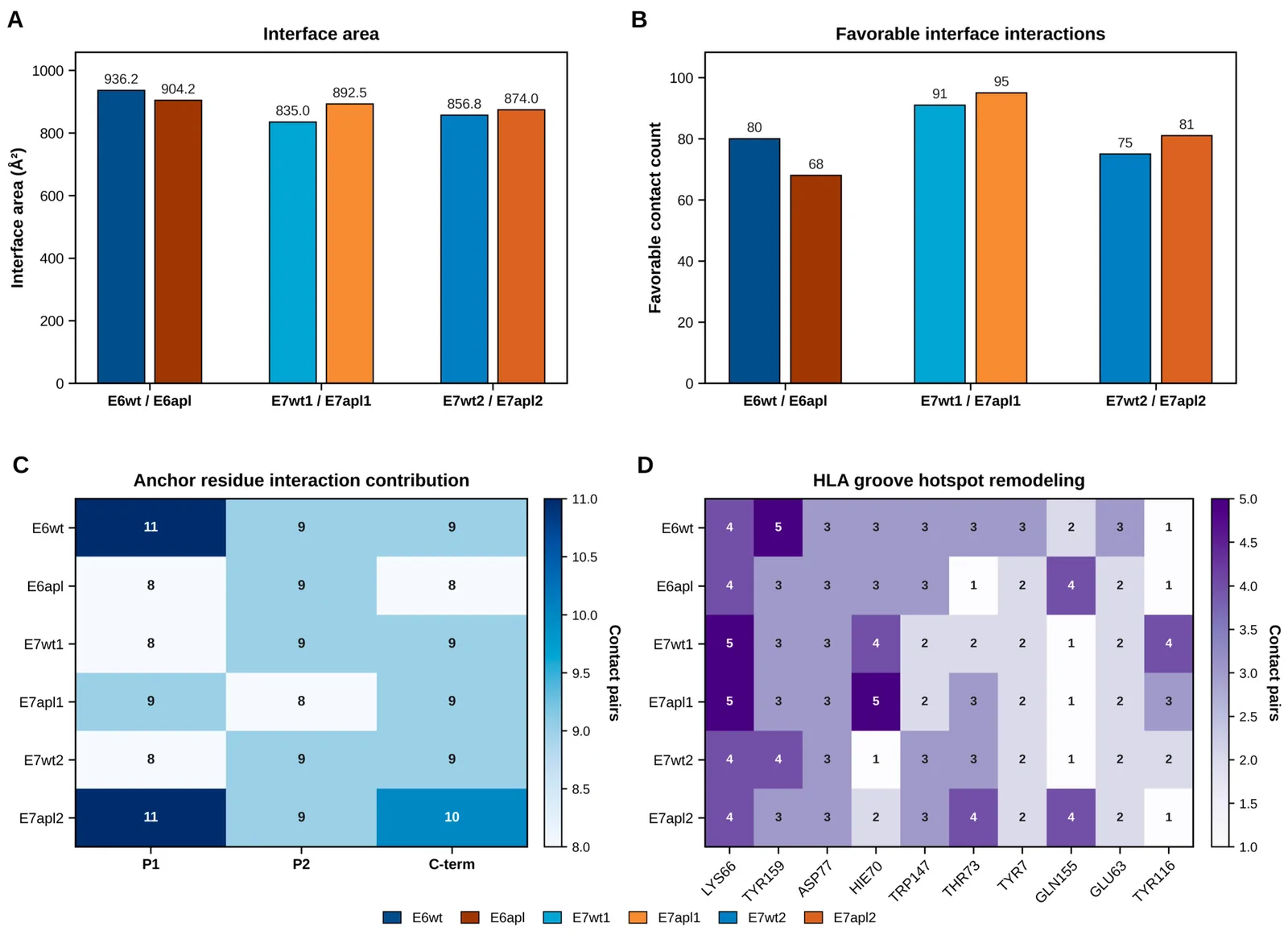
COCOMAPS 2.0 analysis reveals residue-level remodeling of peptide–HLA-A*02:01 interaction networks induced by APL substitutions. Molecular interaction patterns between HPV16 E6/E7-derived peptides and HLA-A*02:01 were characterized using COCOMAPS 2.0 analysis based on representative structures extracted from molecular dynamics simulations. (**A**) Comparison of buried interface areas between wild-type (WT) and altered peptide ligand (APL) peptide–HLA-A*02:01 complexes. APL substitutions induced distinct interface rearrangements without uniformly increasing the overall buried interface area. (**B**) Quantification of favorable interfacial interactions identified by COCOMAPS 2.0, including hydrogen bonds, CH–O/N bonds, polar and apolar van der Waals contacts, and other stabilizing interactions. E7-derived APLs exhibited increased favorable interaction counts compared with their corresponding WT peptides, whereas E6apl displayed altered interaction composition rather than an overall increase in favorable contacts. (**C**) Contribution of peptide anchor residues (P1, P2, and C-terminal residues) to HLA-A02:01 interface interactions. APL substitutions modified anchor residue engagement patterns, consistent with the rational design strategy targeting HLA-A02:01 anchor preferences. (**D**) Heatmap visualization of HLA-A*02:01 groove hotspot residue interactions. Contact frequencies involving key HLA residues, including Lys66, Tyr159, Asp77, His70, Trp147, Glu63, and Tyr116, demonstrate redistribution of peptide-contact networks following APL modification.

To further characterize how anchor-residue substitutions influenced peptide–HLA-A*02:01 interactions, COCOMAPS 2.0 analysis was performed using representative structures extracted from the equilibrated MD trajectories of the six peptide–HLA complexes (E6wt/E6apl, E7wt1/E7apl1, and E7wt2/E7apl2) (Figure 8). This analysis complemented the MD-based evaluation of peptide conformational properties by providing additional information on interface architecture, residue-level contact patterns, and HLA groove hotspot interactions.

Comparison of the buried interface area showed that APL substitutions induced distinct peptide–HLA interface rearrangements without uniformly increasing the overall contact surface (Figure 8A). E6apl displayed a slightly reduced interface area compared with E6wt (904.2 Å^2^ versus 936.2 Å^2^), whereas E7apl1 and E7apl2 showed increased interface areas compared with their corresponding WT peptides (892.5 Å^2^ versus 835.0 Å^2^ for E7wt1/E7apl1; 874.0 Å^2^ versus 856.8 Å^2^ for E7wt2/E7apl2). These results indicate that enhanced peptide presentation is not simply associated with expansion of the buried interface area, but rather involves specific rearrangement of local peptide–HLA interaction patterns.

Analysis of favorable interfacial interactions further revealed distinct remodeling patterns among APL variants (Figure 8B). E7apl1 and E7apl2 exhibited increased favorable interaction counts compared with their WT counterparts (95 versus 91 and 81 versus 75, respectively). In contrast, E6apl showed reduced total favorable interactions compared with E6wt (68 versus 80), suggesting that functional improvement of E6apl was associated with altered interaction composition rather than a simple increase in the number of favorable contacts.

Residue-level contribution analysis demonstrated that APL substitutions modified peptide anchor residue engagement at the P1, P2, and C-terminal positions (Figure 8C). Notably, E7apl2 showed increased contributions at the P1 and C-terminal positions compared with E7wt2, consistent with the design strategy targeting canonical HLA-A*02:01 anchor preferences. Similar redistribution of anchor residue contributions was observed in other APL complexes, indicating that sequence optimization altered local peptide–HLA interaction patterns.

HLA groove hotspot analysis further demonstrated differential redistribution of peptide-contact networks following APL modification (Figure 8D). Several HLA-A*02:01 residues involved in peptide recognition, including Lys66, Tyr159, Asp77, His70, Trp147, Glu63, and Tyr116, exhibited altered contact patterns among WT and APL complexes. Detailed interaction-type distributions, peptide residue contributions, and WT/APL differential interaction profiles are summarized in Supplementary Figure S3.

Together, COCOMAPS 2.0 analysis provided additional structural characterization of APL-induced peptide–HLA-A*02:01 interface remodeling. These findings complement the MD-derived conformational analyses and suggest that optimized APL design involves selective modulation of local molecular interaction networks rather than simply maximizing overall peptide–HLA contact strength.

## 4. Discussion

### 4.1. An Integrated Dry–Wet Platform Enables Functional meHPV-CTL Generation

We established an integrated dry–wet workflow that translates in silico epitope predictions into a functional multi-epitope CTL formulation (Figure 1). Initial wild-type (WT) epitope selection integrated orthogonal lines of evidence, including predicted HLA presentation, curated immunogenicity databases, and clinical literature support, which may help reduce the false-positive rates associated with individual prediction approaches [12].

Our APL design strategy was intentionally conservative, restricting substitutions to the canonical HLA-A*02:01 anchor positions (P1, P2, and PΩ). By preserving central residues potentially involved in TCR recognition, this rational design aimed to improve peptide–MHC stability while minimizing alterations to the putative TCR-contacting region [11].

Following this workflow, the resulting six-peptide formulation (E6wt, E7wt1, E7wt2, E6apl, E7apl2, and E7apl3) enabled the generated polyclonal meHPV-CTL populations capable of potent, HLA class I-restricted cytotoxicity against HPV16 E7-expressing cervical carcinoma cells, reaching >74% specific lysis at peak effector-to-target ratios (Figure 6).

Considered together, these results support the utility of an integrated dry–wet workflow for epitope prioritization beyond purely in silico selection strategies. More importantly, the functional comparison between WT and APL candidates highlights the necessity of combining computational prediction with experimental validation for identifying functionally competent epitopes. This study provides a framework for the rational development of multi-epitope HPV16-directed cellular immunotherapeutic approaches.

### 4.2. The Binding–Immunogenicity Dissociation Reveals the Importance of Functional Validation Beyond Binding Prediction

A key observation of this study is the dissociation between predicted HLA-A*02:01 binding affinity and functional T-cell responses, illustrated by the E7apl1 epitope. Although E7apl1 was predicted to be a strong binder (Table 1) and supported CD8^+^ T-cell expansion comparable to its wild-type counterpart (Figure 3), it induced reduced IFN-γ secretion in ELISpot assays relative to E7wt1 (Figure 4). These findings indicate that enhanced peptide–HLA binding and T-cell proliferation do not necessarily translate into proportional effector functionality, highlighting the importance of functional evaluation beyond affinity-based prediction.

Molecular dynamics simulations provided structural insight into this functional divergence (Figure 7, Table 2). Across all WT/APL pairs, MM-GBSA-derived binding free energies were broadly comparable, suggesting that overall thermodynamic stability alone may not fully explain the observed functional differences. These estimates provide relative stability measures rather than absolute predictors of biological activity, as they do not explicitly account for direct TCR engagement, entropic contributions, or conformational heterogeneity of the immune synapse.

Within this framework, the E7apl1 substitution (L1Y) was associated with altered local peptide dynamics within the HLA-A*02:01 groove. Per-residue RMSF analysis indicated a more constrained conformational profile compared with E7wt1, including changes involving solvent-exposed peptide regions. However, these computational observations should be interpreted as structural hypotheses rather than direct evidence of altered TCR recognition, as pMHC–TCR interactions were not experimentally characterized in this study.

Complementary COCOMAPS 2.0 analysis further demonstrated that APL substitutions reshaped residue-level peptide–HLA interaction patterns, including changes in buried interface properties, favorable interaction profiles, anchor residue contributions, and HLA groove hotspot contacts (Figure 8 and Figure S3). These results suggest that functional differences among APLs may involve complex alterations in peptide–HLA interaction landscapes rather than simple enhancement of overall binding strength.

In contrast, E6apl provides a complementary observation to this trend. Despite exhibiting the weakest predicted binding free energy among all simulated complexes (ΔG_bind_ = −73.59 kcal/mol), it demonstrated robust functional immunogenicity in vitro. This observation further supports the concept that optimal CTL activation is not solely determined by thermodynamic binding strength, but may depend on the balance between peptide–MHC stability, conformational properties, and downstream immune recognition processes.

Collectively, these findings support a model in which stable peptide–MHC binding represents an important but insufficient factor for productive CD8^+^ T-cell activation. Although the integrated MD and interface analyses provide additional structural perspectives on APL behavior, direct experimental characterization of pMHC–TCR interactions remains necessary to further validate the mechanisms underlying functional peptide recognition.

### 4.3. Translational Significance of the Mixed WT/APL Pool

The final vaccine formulation integrates anchor-optimized altered peptide ligands (APLs) with their corresponding parental wild-type (WT) epitopes, and is designed to combine two complementary immunological functions within a single multi-epitope framework. WT peptides preserve native sequence fidelity for tumor-associated antigen recognition, whereas APLs enhance HLA-A*02:01 binding stability and may improve CD8^+^ T-cell priming efficiency [10]. This dual-component design combines antigen sequence fidelity with improved MHC presentation properties to support polyclonal CTL induction.

This WT/APL hybrid strategy may address a key limitation of purely optimized epitope designs, namely the potential loss of native antigen recognition due to excessive sequence modification. Conversely, APL inclusion may partially compensate for the relatively weak immunogenicity of certain WT epitopes by enhancing peptide–MHC stability and improving the likelihood of productive T-cell priming. In this context, the formulation provides complementary antigen sources with distinct sequence and presentation characteristics within a multi-epitope framework.

Compared with established HPV16 therapeutic vaccine platforms—including synthetic long-peptide vaccines [41], DNA-based vaccines [42], liposomal peptide nanoparticle systems [4], and peptide-adjuvant formulations [43]—the present strategy emphasizes a rational, structure-guided epitope engineering pipeline. Rather than relying exclusively on empirical peptide selection, the workflow integrates computational screening, structural refinement, and functional validation prior to multi-epitope assembly. This sequential dry–wet design may improve epitope prioritization efficiency and reduces dependence on trial-and-error immunogenicity testing.

Notably, simultaneous targeting of multiple HPV16 E6/E7 determinants may broaden CD8^+^ T-cell specificity and reduce the risk of immune escape associated with antigen loss or epitope variation [44]. Consistent with this rationale, previous studies have demonstrated that multi-epitope CTL vaccines can induce robust IFN-γ responses and provide tumor protection in the TC-1 model, supporting further evaluation of multi-determinant strategies in future in vivo preclinical studies [45]. Because the selected epitopes are derived from structurally constrained and functionally important regions of HPV16 oncoproteins, they may show reduced tolerance to sequence variation. In the present study, additional HPV16 sequence conservation and HLA-A*02:01 population coverage analyses further supported the potential translational relevance of the selected epitopes and provided additional evidence for their applicability in HLA-A*02:01-positive populations [46].

### 4.4. Limitations

Several limitations of this study should be considered when interpreting the results. First, functional validation was performed using a limited number of HLA-A*02:01-positive healthy donors. Although consistent response patterns were observed across experiments, donor-to-donor variability in antigen responsiveness and immune background may influence the magnitude of peptide-specific T-cell responses.

Second, T-cell receptor (TCR) repertoire diversity and clonality were not evaluated in the present study. Given the important role of TCR composition in determining epitope-specific expansion and effector function, the absence of clonal-level analysis limits further mechanistic interpretation of the observed functional differences among peptide-stimulated populations.

Third, although Mock-T cells were included as a shared effector control in cytotoxicity assays to evaluate background cytotoxic activity, donor-level tracking was not performed for this control population. Therefore, comparisons involving Mock-T should be interpreted primarily as relative assessments of background killing rather than donor-matched quantitative measurements.

Fourth, all functional assays were performed in vitro using peptide-pulsed dendritic cell stimulation systems. Although this platform enables controlled evaluation of antigen-specific CD8^+^ T-cell responses, it does not fully reproduce endogenous antigen processing, tumor microenvironmental suppression, or the complexity of in vivo immune regulation. Therefore, further validation in appropriate preclinical models will be required to evaluate the translational potential of the multi-epitope formulation.

Fifth, the computational analyses, including molecular dynamics simulations, MM-GBSA calculations, and COCOMAPS 2.0-based interface characterization, provide relative structural and energetic insights into peptide–HLA-A*02:01 interactions. Although block-averaged analyses supported the stability of the major structural trends observed in the simulations, these approaches do not fully capture conformational entropy or direct pMHC–TCR interaction dynamics. Therefore, the structural interpretations presented here should be considered hypothesis-generating and require further validation through pMHC–TCR structural studies or biophysical measurements.

In summary, this study should be regarded as a proof-of-concept demonstration of an integrated immunoinformatics-to-functional validation framework for HPV16 E6/E7 epitope optimization. Future studies incorporating larger and more diverse donor cohorts, TCR repertoire profiling, in vivo validation, and direct characterization of pMHC–TCR interactions will be important for further evaluating and advancing this strategy toward translational application.

## 5. Conclusions

This study integrates immunoinformatics-guided epitope engineering with systematic experimental validation to establish an integrated workflow for the design and functional evaluation of HPV16 E6/E7-derived altered peptide ligands (APLs). Using this pipeline, we identified functionally validated anchor-optimized epitopes and constructed a six-peptide formulation composed of three wild-type (WT) epitopes and three optimized APLs.

The resulting formulation successfully generated polyclonal, multi-epitope HPV-specific CD8^+^ T-cell populations (meHPV-CTLs) that exhibited potent, HLA class I-restricted, and target-specific cytotoxicity against HPV16 E7-expressing cervical carcinoma cells. These findings demonstrate the feasibility of combining native and optimized epitopes within a minimal multi-epitope formulation to induce functionally active CTL responses.

Importantly, functional analyses revealed a dissociation between predicted peptide–HLA binding affinity and effector T-cell activity, demonstrating that enhanced HLA binding alone is insufficient to predict optimal immunogenic outcomes. This observation highlights the necessity of integrating structural prediction with downstream immunological validation in rational APL design.

In conclusion, this work establishes an integrated immunoinformatics-to-function framework that links epitope prediction, anchor-residue optimization, and cellular immune validation for the development of multi-epitope CTL-based immunotherapies. These results provide a conceptual and methodological foundation for the future development of HPV-targeted cellular immunotherapies, while future studies incorporating in vivo validation and direct characterization of pMHC–TCR interactions will be required to further evaluate therapeutic potential.

## Figures and Tables

**Table 1:** In silico characterization and prioritization of HPV16 E6/E7-derived wild-type epitopes and altered peptide ligand (APL) candidates restricted by HLA-A*02:01.

Protein	Epitope ID	Peptide Sequence	WT Sequence	Substitution	HLA	Position	Immunogenicity	Antigenicity	Toxicity	Presentation Score	%Rank (EL)	Affinity (nM)	Binding Level
E6	E6wt	TIHDIILECV	—	—	A*02:01	29–38	−0.008	1.00	Non-Toxin	0.286	0.990	329.33	WB
E6	E6apl	YLHDIILECV	TIHDIILECV	T1→Y; I2→L	A*02:01	29–38	0.111	1.00	Non-Toxin	0.873	0.024	6.21	SB
E7	E7wt1	LLMGTLGIV	—	—	A*02:01	82–90	−0.008	0.66	Non-Toxin	0.238	1.258	12.59	WB
E7	E7apl1	YLMGTLGIV	LLMGTLGIV	L1→Y	A*02:01	82–90	0.335	0.66	Non-Toxin	0.582	0.298	4.24	SB
E7	E7wt2	YMLDLQPETT	—	—	A*02:01	11–20	−0.008	0.66	Non-Toxin	0.164	1.442	207.32	WB
E7	E7apl2	YMLDLQPETV	YMLDLQPETT	T10→V	A*02:01	11–20	0.335	0.66	Non-Toxin	0.873	0.063	6.63	SB
E7	E7apl3	YLLDLQPETV	YMLDLQPETT	M2→L; T10→V	A*02:01	11–20	0.111	0.66	Non-Toxin	0.947	0.025	6.14	SB

WB, weak binder; SB, strong binder; — not applicable.

**Table 2:** MM-GBSA binding free energy decomposition for HPV16 E6/E7-derived wild-type (WT) and altered peptide ligand (APL) complexes with HLA-A*02:01.

Peptide	ΔE_vdW_ (kcal/mol)	ΔE_elec_ (kcal/mol)	ΔG_polar_ (kcal/mol)	ΔG_nonpolar_ (kcal/mol)	ΔG_bind_ (kcal/mol)
E6wt (10-mer)	−86.74 ± 6.71	−185.56 ± 21.78	201.77 ± 20.52	−13.60 ± 0.66	−84.14 ± 8.80
E6apl (10-mer)	−82.44 ± 5.53	−270.22 ± 26.73	291.97 ± 22.16	−12.91 ± 0.49	−73.59 ± 8.83
E7wt1 (9-mer)	−83.87 ± 4.91	−257.49 ± 36.12	278.36 ± 29.71	−12.46 ± 0.39	−75.47 ± 9.20
E7apl1 (9-mer)	−90.46 ± 4.40	−240.53 ± 27.31	263.77 ± 23.09	−12.99 ± 0.40	−80.21 ± 7.12
E7wt2 (10-mer)	−79.43 ± 6.65	−263.58 ± 26.96	277.50 ± 22.48	−12.59 ± 0.55	−78.09 ± 9.25
E7apl2 (10-mer)	−80.21 ± 5.95	−299.54 ± 38.91	310.46 ± 34.03	−12.95 ± 0.54	−82.23 ± 9.53

WT, wild-type; APL, altered peptide ligand. Energy terms: ΔE_vdW_, van der Waals; ΔE_elec_, electrostatic; ΔG_polar_, polar solvation (generalized Born); ΔG_nonpolar_, nonpolar solvation (surface area term); ΔG_bind_, total predicted binding free energy. Values are reported as mean ± standard deviation of per-frame MM-GBSA estimates using a single-trajectory approach. A total of 600 frames were extracted at 10-frame intervals from the equilibrated 20–50 ns portion of the 50 ns trajectory (Onufriev GB-OBC II model, igb = 5; 0.15 M salt). Conformational entropy (−TΔS) was not included. Nomenclature follows Table 1. MM-GBSA values should be interpreted as relative indicators of binding stability within closely related ligand sets.

## Data Availability

Data Availability Statement: The original data and code presented in this study are openly available in Zenodo (https://doi.org/10.5281/zenodo.21129910), and can also be accessed via the associated GitHub repository (https://github.com/dongdian1022/hpv16-immunoinformatics-apl-ctl). The repository contains the processed datasets, in silico prediction outputs, molecular dynamics and MM-GBSA analysis results, the source data underlying all figures, and all custom analysis and plotting scripts utilized in this study. Further inquiries can be directed to the corresponding author.

## Supplementary Materials

The following supporting information can be downloaded at: https://www.mdpi.com/article/10.3390/vaccines14080715/s1, Figure S1: Full-length conservation of HPV16 E6 and E7 proteins across representative viral sublineages; Figure S2: Conservation of HPV16 E6/E7-derived wild-type epitopes and altered peptide ligand (APL) candidates; Figure S3: Detailed COCOMAPS 2.0-based characterization of peptide–HLA-A*02:01 interaction profiles; Figure S4: Block-averaged validation of molecular dynamics simulation stability; Figure S5: IEDB-based population coverage analysis of the selected HLA-A*02:01-restricted HPV16 epitopes; Table S1: Conservation of HPV16 residues targeted for altered peptide ligand (APL) optimization; Table S2: Overview of peptide evaluation, experimental validation, and final formulation selection; Table S3: Summary of IEDB population coverage analysis for the selected HLA-A*02:01-restricted HPV16 epitopes.
