# Supplementary material for: Immunoinformatics-Driven Rational Design of Altered Peptide Ligands and In Vitro Validation of a Multi-Epitope CTL Formulation Targeting HPV16 E6/E7
Source: Vaccines (Basel). 2026 Aug 20;14(8):715. doi: 10.3390/vaccines14080715 (PMC13517650; doi:10.3390/vaccines14080715)
Supplement: Supplementary file 1 [file vaccines-14-00715-s001.zip › Figure S1. Full-length conservation of HPV16 E6 and E7 proteins across representative viral sublineages..pdf]

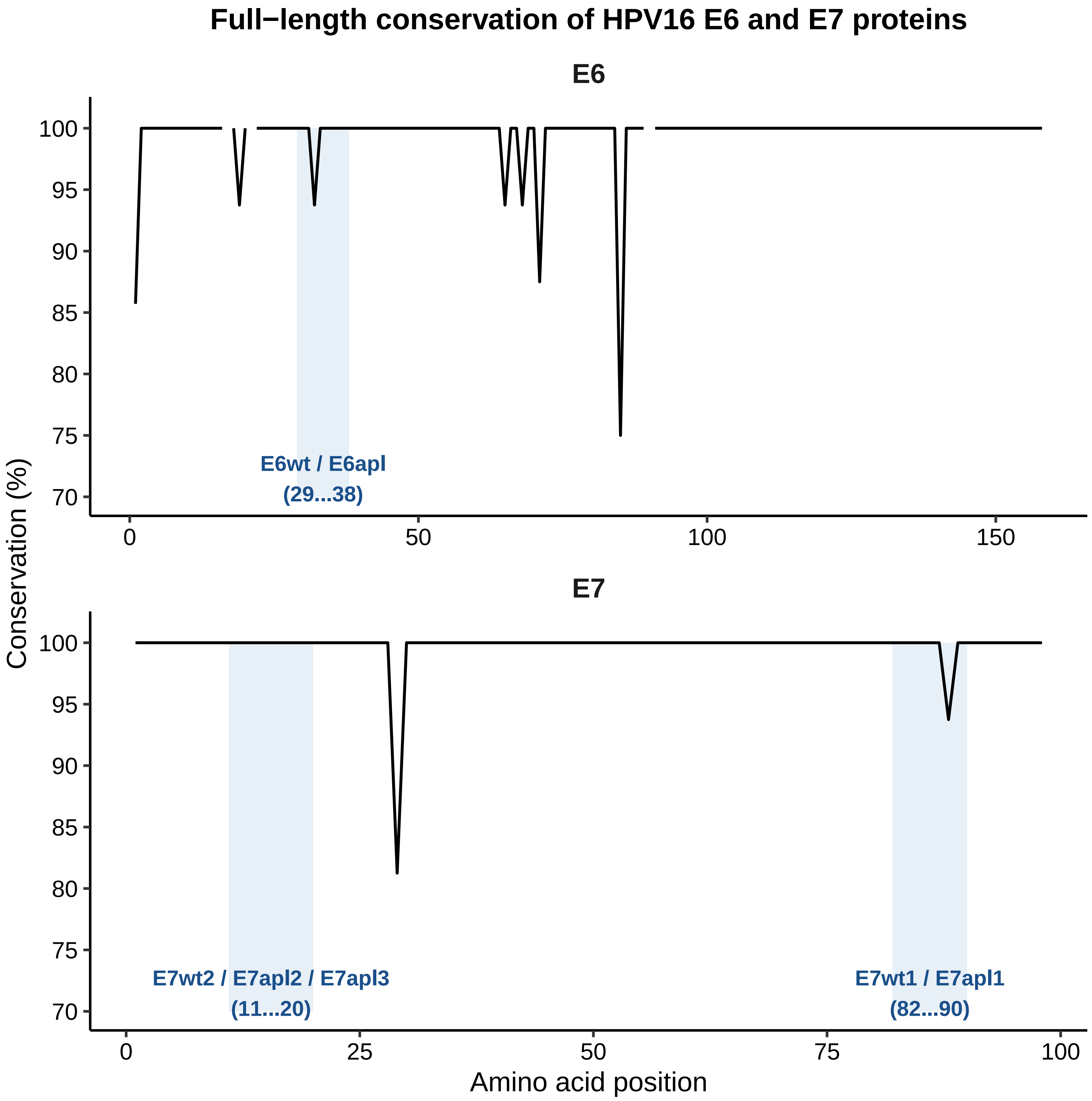

Full−length conservation of HPV16 E6 and E7 proteins
E6
E6wt / E6apl
(29...38)
E7
E7wt2 / E7apl2 / E7apl3
(11...20)
E7wt1 / E7apl1
(82...90)
Conservation (%)
Amino acid position
100
95
90
85
80
75
70
0
50
100
150
0
25
50
75
100
