# Supplementary material for: Immunoinformatics-Driven Rational Design of Altered Peptide Ligands and In Vitro Validation of a Multi-Epitope CTL Formulation Targeting HPV16 E6/E7
Source: Vaccines (Basel). 2026 Aug 20;14(8):715. doi: 10.3390/vaccines14080715 (PMC13517650; doi:10.3390/vaccines14080715)
Supplement: Supplementary file 1 [file vaccines-14-00715-s001.zip › Figure S2. Conservation of HPV16 E6E7-derived wild-type epitopes and altered peptide ligand (APL) candidates..pdf]

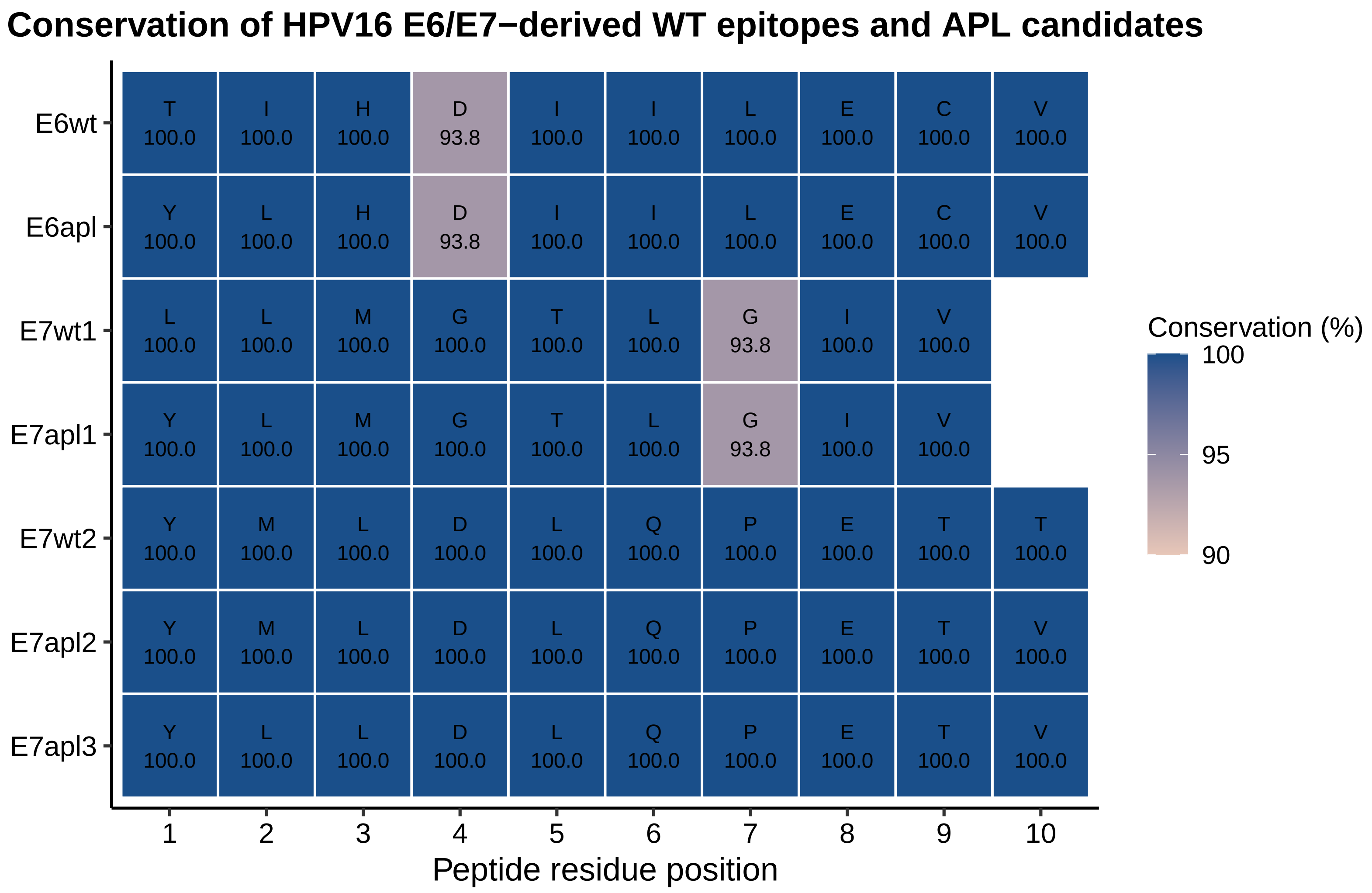

Conservation of HPV16 E6/E7−derived WT epitopes and APL candidates
E6wt
E6apl
E7wt1
E7apl1
E7wt2
E7apl2
E7apl3
T I H D I I L E C V
100.0 100.0 100.0 93.8 100.0 100.0 100.0 100.0 100.0 100.0
Y L H D I I L E C V
100.0 100.0 100.0 93.8 100.0 100.0 100.0 100.0 100.0 100.0
L L M G T L G I V
100.0 100.0 100.0 100.0 100.0 100.0 93.8 100.0 100.0
Y L M G T L G I V
100.0 100.0 100.0 100.0 100.0 100.0 93.8 100.0 100.0
Y M L D L Q P E T T
100.0 100.0 100.0 100.0 100.0 100.0 100.0 100.0 100.0 100.0
Y M L D L Q P E T V
100.0 100.0 100.0 100.0 100.0 100.0 100.0 100.0 100.0 100.0
Y L L D L Q P E T V
100.0 100.0 100.0 100.0 100.0 100.0 100.0 100.0 100.0 100.0
1 2 3 4 5 6 7 8 9 10
Peptide residue position
Conservation (%)
100
95
90
