# Supplementary material for: Immunoinformatics-Driven Rational Design of Altered Peptide Ligands and In Vitro Validation of a Multi-Epitope CTL Formulation Targeting HPV16 E6/E7
Source: Vaccines (Basel). 2026 Aug 20;14(8):715. doi: 10.3390/vaccines14080715 (PMC13517650; doi:10.3390/vaccines14080715)
Supplement: Supplementary file 1 [file vaccines-14-00715-s001.zip › Figure S3. Detailed COCOMAPS2.0-based characterization of peptide–HLA-A0201 interaction profiles.pdf]

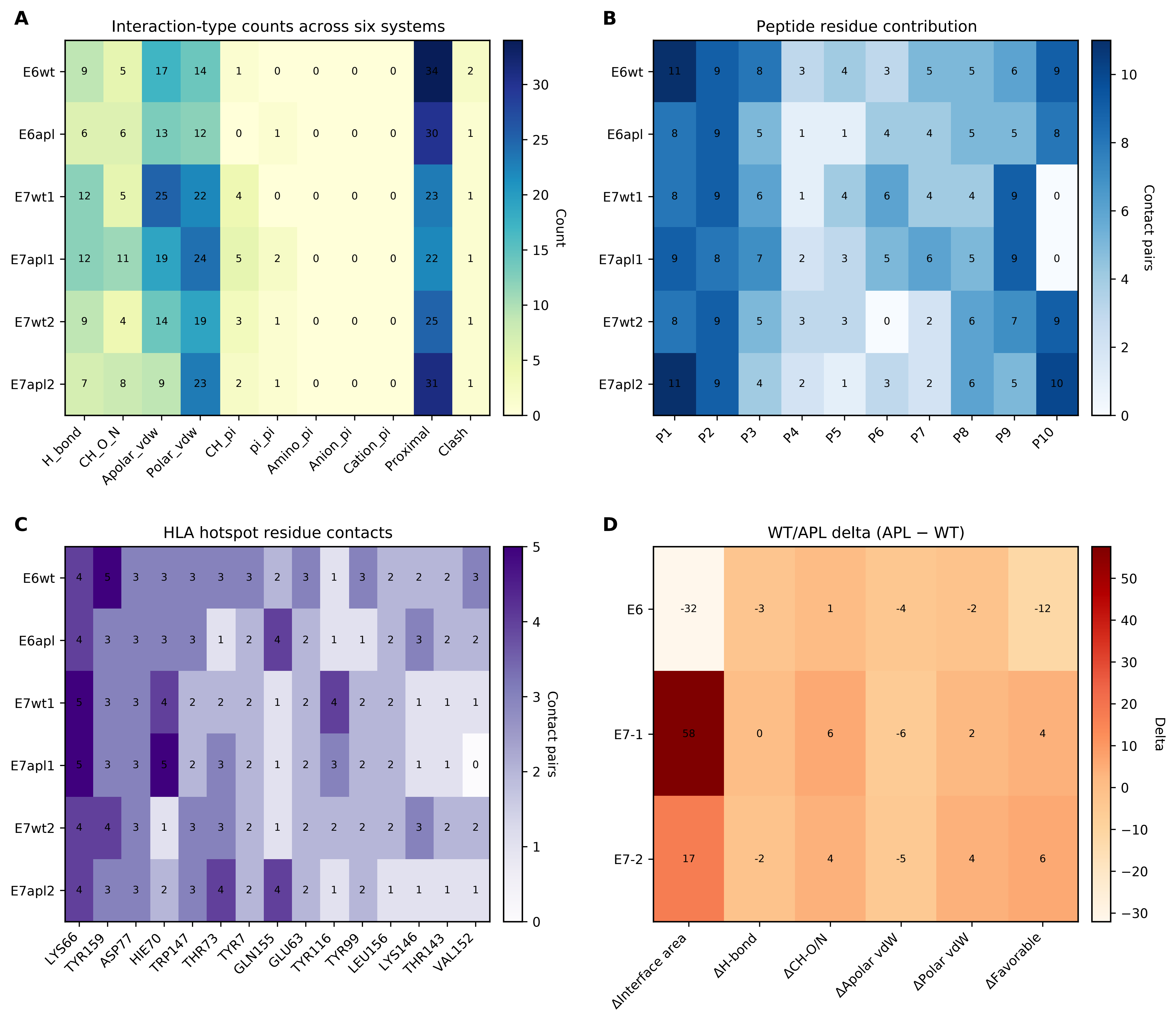
A
Interaction-type counts across six systems
H_bond CH_O_N Apolar_vdw Polar_vdw CH_pi pi_pi Amino_pi Anion_pi Cation_pi Proximal Clash
E6wt 9 5 17 14 1 0 0 0 0 34 2
E6apl 6 6 13 12 0 1 0 0 0 30 1
E7wt1 12 5 25 22 4 0 0 0 0 23 1
E7apl1 12 11 19 24 5 2 0 0 0 22 1
E7wt2 9 4 14 19 3 1 0 0 0 25 1
E7apl2 7 8 9 23 2 1 0 0 0 31 1
Count
0 5 10 15 20 25 30
B
Peptide residue contribution
P1 P2 P3 P4 P5 P6 P7 P8 P9 P10
E6wt 11 9 8 3 4 3 5 5 6 9
E6apl 8 9 5 1 1 4 4 5 5 8
E7wt1 8 9 6 1 4 6 4 4 9 0
E7apl1 9 8 7 2 3 5 6 5 9 0
E7wt2 8 9 5 3 3 0 2 6 7 9
E7apl2 11 9 4 2 1 3 2 6 5 10
Contact pairs
0 2 4 6 8 10
C
HLA hotspot residue contacts
LYS66 TYR159 ASP77 HIE70 TRP147 THR73 TYR7 GLN155 GLU63 TYR116 TYR99 LEU156 LYS146 THR143 VAL152
E6wt 4 5 3 3 3 3 3 2 3 1 3 2 2 2 3
E6apl 4 3 3 3 3 1 2 4 2 1 1 2 3 2 2
E7wt1 5 3 3 4 2 2 2 1 2 4 2 2 1 1 1
E7apl1 5 3 3 5 2 3 2 1 2 3 2 2 1 1 0
E7wt2 4 4 3 1 3 3 2 1 2 2 2 2 3 2 2
E7apl2 4 3 3 2 3 4 2 4 2 1 2 1 1 1 1
Contact pairs
0 1 2 3 4 5
D
WT/APL delta (APL − WT)
ΔInterface area ΔH-bond ΔCH-O/N ΔApolar vdW ΔPolar vdW ΔFavorable
E6 -32 -3 1 -4 -2 -12
E7-1 58 0 6 -6 2 4
E7-2 17 -2 4 -5 4 6
Delta
−30 −20 −10 0 10 20 30 40 50
