# Supplementary material for: Immunoinformatics-Driven Rational Design of Altered Peptide Ligands and In Vitro Validation of a Multi-Epitope CTL Formulation Targeting HPV16 E6/E7
Source: Vaccines (Basel). 2026 Aug 20;14(8):715. doi: 10.3390/vaccines14080715 (PMC13517650; doi:10.3390/vaccines14080715)
Supplement: Supplementary file 1 [file vaccines-14-00715-s001.zip › Figure S4. Block-averaged validation of molecular dynamics simulation stability..pdf]

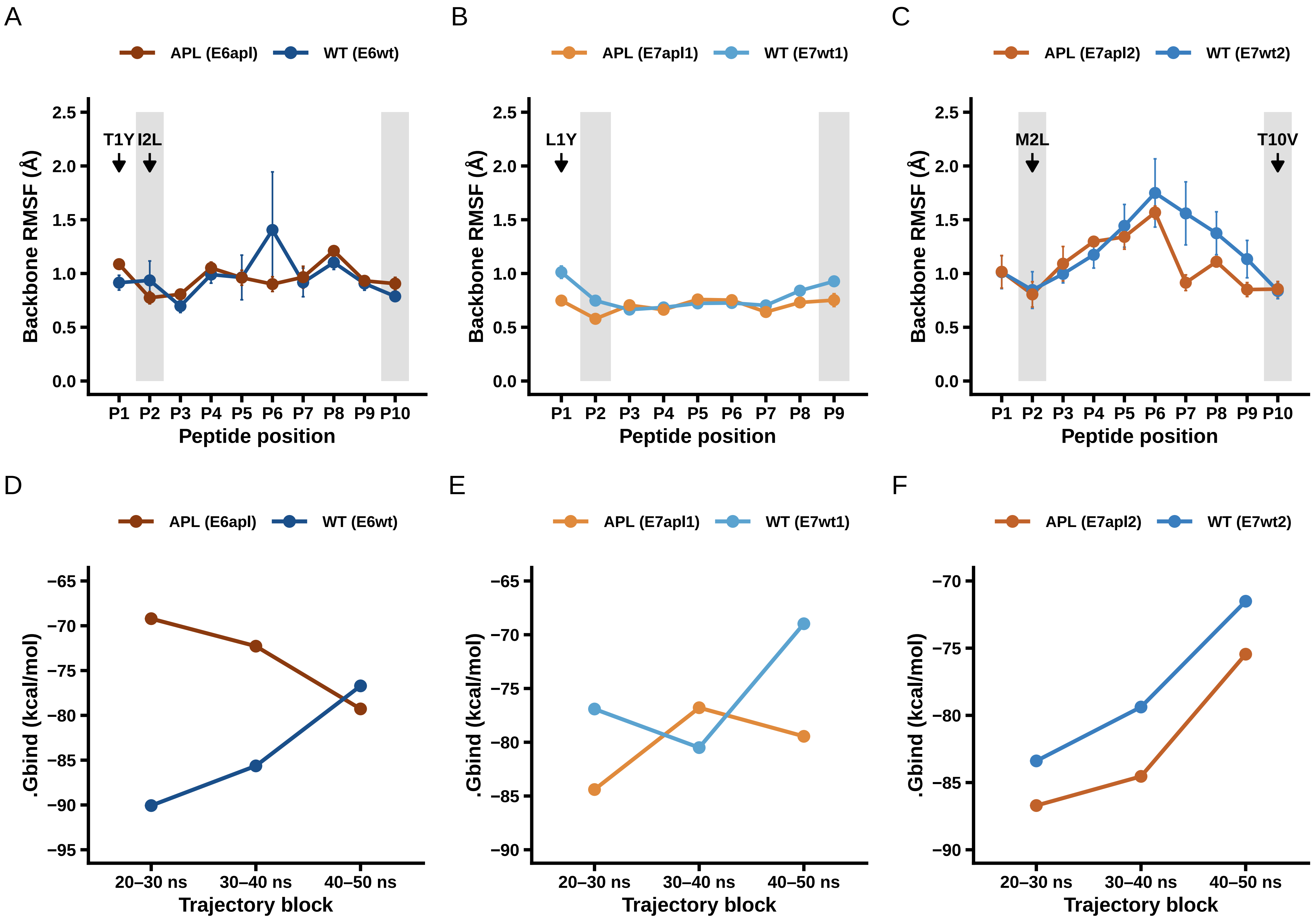

A
APL (E6apl)
WT (E6wt)
T1Y
I2L
Backbone RMSF (Å)
2.5
2.0
1.5
1.0
0.5
0.0
P1 P2 P3 P4 P5 P6 P7 P8 P9 P10
Peptide position
B
APL (E7apl1)
WT (E7wt1)
L1Y
Backbone RMSF (Å)
P1 P2 P3 P4 P5 P6 P7 P8 P9
Peptide position
C
APL (E7apl2)
WT (E7wt2)
M2L
T10V
Backbone RMSF (Å)
P1 P2 P3 P4 P5 P6 P7 P8 P9 P10
Peptide position
D
APL (E6apl)
WT (E6wt)
.Gbind (kcal/mol)
−65
−70
−75
−80
−85
−90
−95
20–30 ns
30–40 ns
40–50 ns
Trajectory block
E
APL (E7apl1)
WT (E7wt1)
.Gbind (kcal/mol)
20–30 ns
30–40 ns
40–50 ns
Trajectory block
F
APL (E7apl2)
WT (E7wt2)
.Gbind (kcal/mol)
20–30 ns
30–40 ns
40–50 ns
Trajectory block
