# Supplementary material for: Immunoinformatics-Driven Rational Design of Altered Peptide Ligands and In Vitro Validation of a Multi-Epitope CTL Formulation Targeting HPV16 E6/E7
Source: Vaccines (Basel). 2026 Aug 20;14(8):715. doi: 10.3390/vaccines14080715 (PMC13517650; doi:10.3390/vaccines14080715)
Supplement: Supplementary file 1 [file vaccines-14-00715-s001.zip › Figure S5. IEDB-based population coverage analysis of the selected HLA-A0201-restricted HPV16 epitopes.pdf]

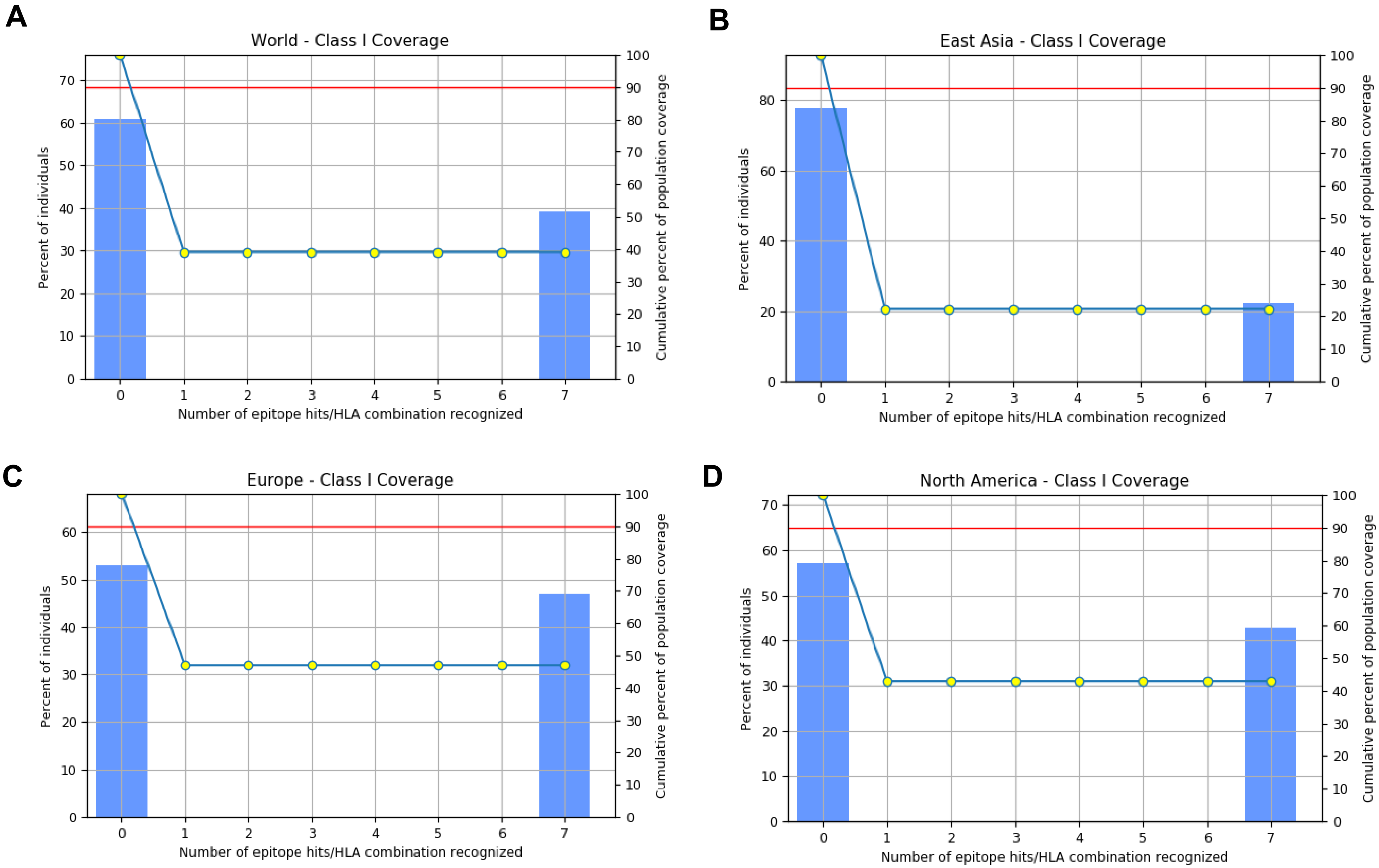
A
World - Class I Coverage
B
East Asia - Class I Coverage
C
Europe - Class I Coverage
D
North America - Class I Coverage
Percent of individuals
Cumulative percent of population coverage
Number of epitope hits/HLA combination recognized
