# Supplementary material for: Immunoinformatics-Driven Rational Design of Altered Peptide Ligands and In Vitro Validation of a Multi-Epitope CTL Formulation Targeting HPV16 E6/E7
Source: Vaccines (Basel). 2026 Aug 20;14(8):715. doi: 10.3390/vaccines14080715 (PMC13517650; doi:10.3390/vaccines14080715)
Supplement: Supplementary file 1 [file vaccines-14-00715-s001.zip › Supplementary Materials.pdf]

Figure S1. Full-length conservation of HPV16 E6 and E7 proteins across representative viral sublineages.

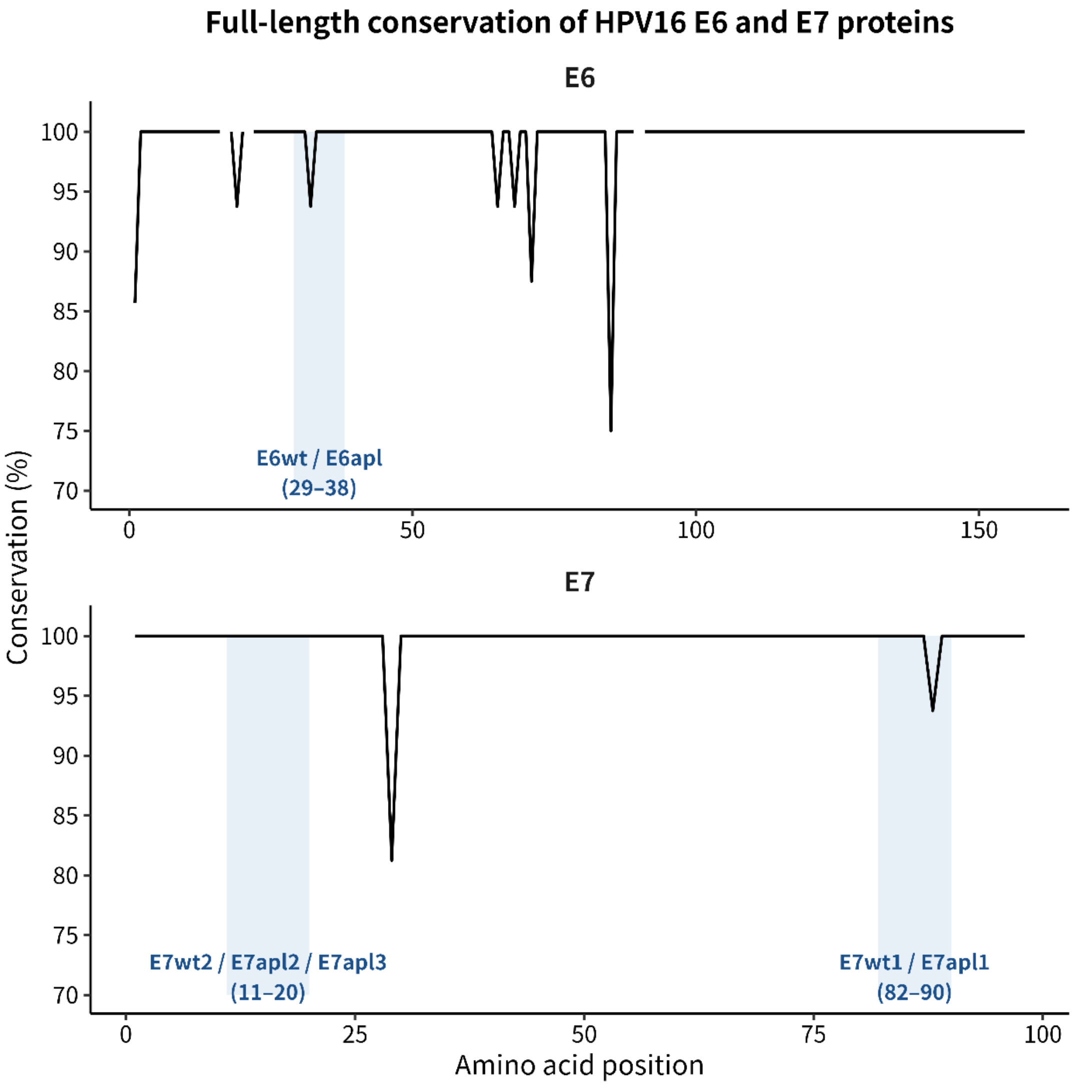

Sequence conservation analysis was performed using amino acid alignments of HPV16 E6 and E7 proteins from 16 representative sublineages (A1–D4). Conservation scores were calculated at each amino acid position based on the frequency of the dominant residue among analyzed variants. The regions corresponding to the experimentally evaluated HPV16-derived epitopes are highlighted, including E6wt/E6apl (E6 residues 29–38), E7wt2/E7apl2/E7apl3 (E7 residues 11–20), and E7wt1/E7apl1 (E7 residues 82–90). The analysis demonstrated that both HPV16 E6 and E7 proteins exhibit high sequence conservation, supporting the selection of these regions for epitope optimization and APL design.

Figure S2. Conservation of HPV16 E6/E7-derived wild-type epitopes and altered peptide ligand (APL) candidates.

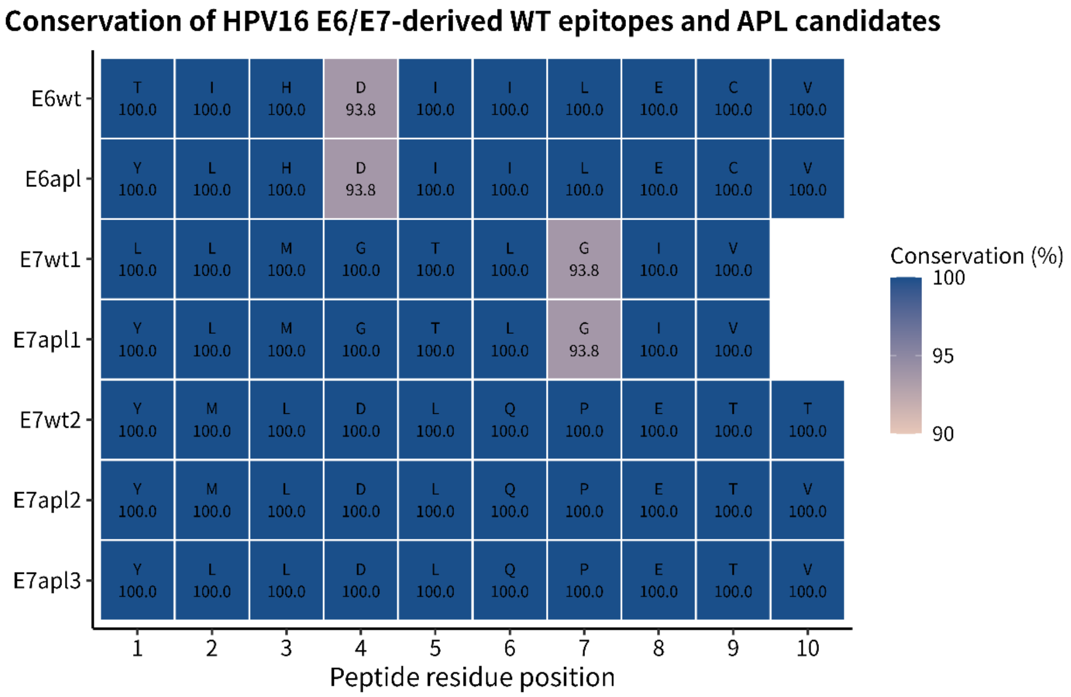

Residue-level conservation of selected HPV16 E6/E7-derived wild-type epitopes and corresponding APL sequences was evaluated across 16 representative HPV16 sublineages. The heatmap displays the conservation percentage of each peptide residue position, with amino acid identities indicated within each cell. The analyzed peptides include E6wt (TIHDIILECV), E6apl (YLHDIILECV), E7wt1 (LLMGTLGIV), E7apl1 (YLMGTLGIV), E7wt2 (YMLDLQPETT), E7apl2 (YMLDLQPETV), and E7apl3 (YLLDLQPETV). All selected epitope regions showed strong conservation, and residues targeted for APL optimization corresponded to highly conserved natural viral positions.

Figure S3. Detailed COCOMAPS 2.0-based characterization of peptide–HLA-A*02:01 interaction profiles.

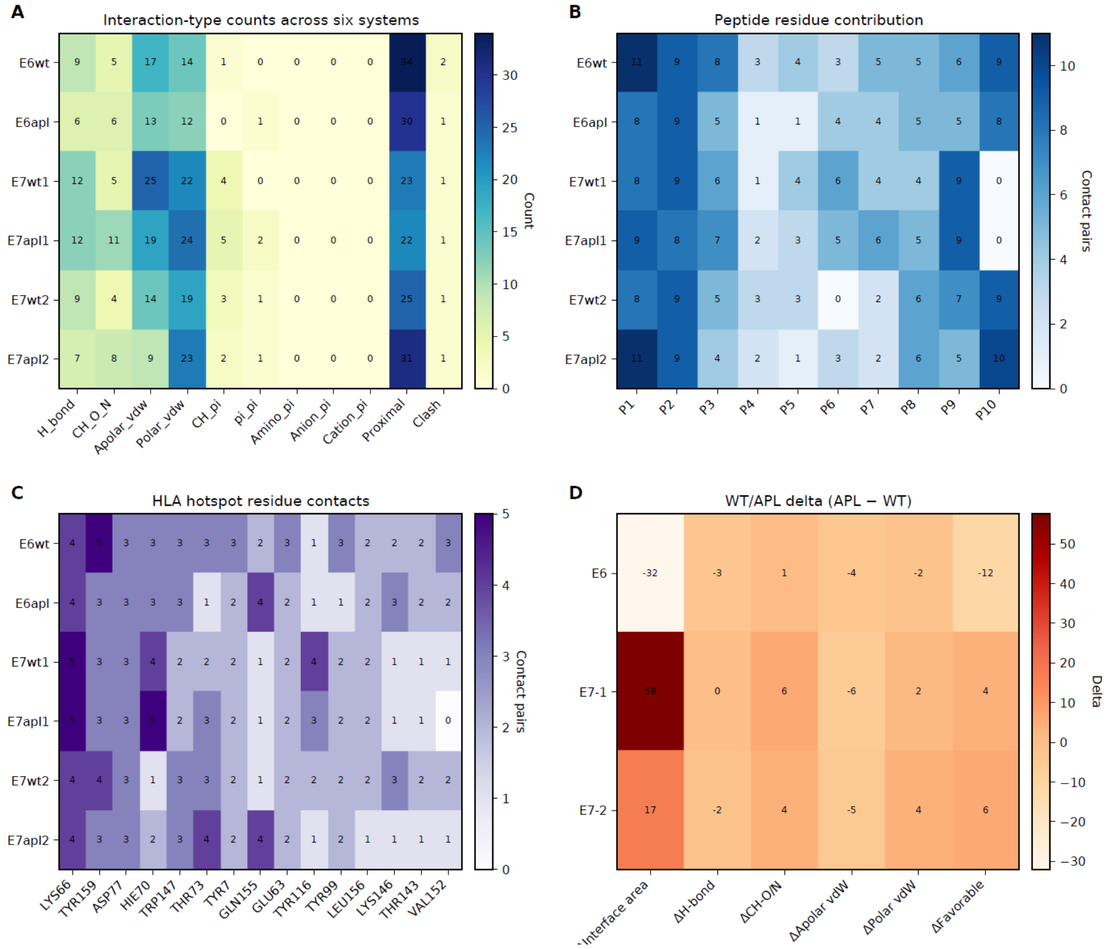

Detailed residue-level interaction analyses of the six simulated WT/APL peptide–HLA-A*02:01 complexes were performed using COCOMAPS2.0. Representative structures extracted from the equilibrated molecular dynamics trajectories were analyzed to characterize interaction composition, peptide residue contributions, and differential WT/APL contact patterns.

(A) Interaction-type profiles across the six peptide–HLA-A*02:01 complexes, including hydrogen bonds, CH–O/N bonds, polar and apolar van der Waals interactions, π-related interactions, proximal contacts, and other interaction categories.

(B) Peptide residue contribution profiles showing the contact contribution of individual peptide positions to the peptide–HLA-A*02:01 interface.

(C) Contact profiles of HLA-A*02:01 groove hotspot residues involved in peptide recognition.

(D) Differential interaction profiles comparing each APL with its corresponding WT complex.

Values represent APL-induced changes calculated as Δ = APL − WT.

Together, these detailed interaction profiles provide complementary information to the global interface measurements presented in Figure 8 and further characterize the molecular consequences of anchor-residue optimization at the peptide–HLA-A*02:01 interface.

Figure S4. Block-averaged validation of molecular dynamics simulation stability.

(A–C) Backbone RMSF analysis performed using three consecutive 10 ns blocks extracted from the equilibrated 20–50 ns trajectories of the E6wt/E6apl, E7wt1/E7apl1, and E7wt2/E7apl2 peptide–HLA-A*02:01 complexes. Mutation positions are indicated above the peptide sequences.

(D–F) Block-averaged MM-GBSA binding free energy profiles calculated from the same trajectory segments. Similar RMSF patterns and broadly consistent ΔGbind trends across the three trajectory blocks support the temporal stability of the major structural and energetic observations over the analyzed 20–50 ns interval.

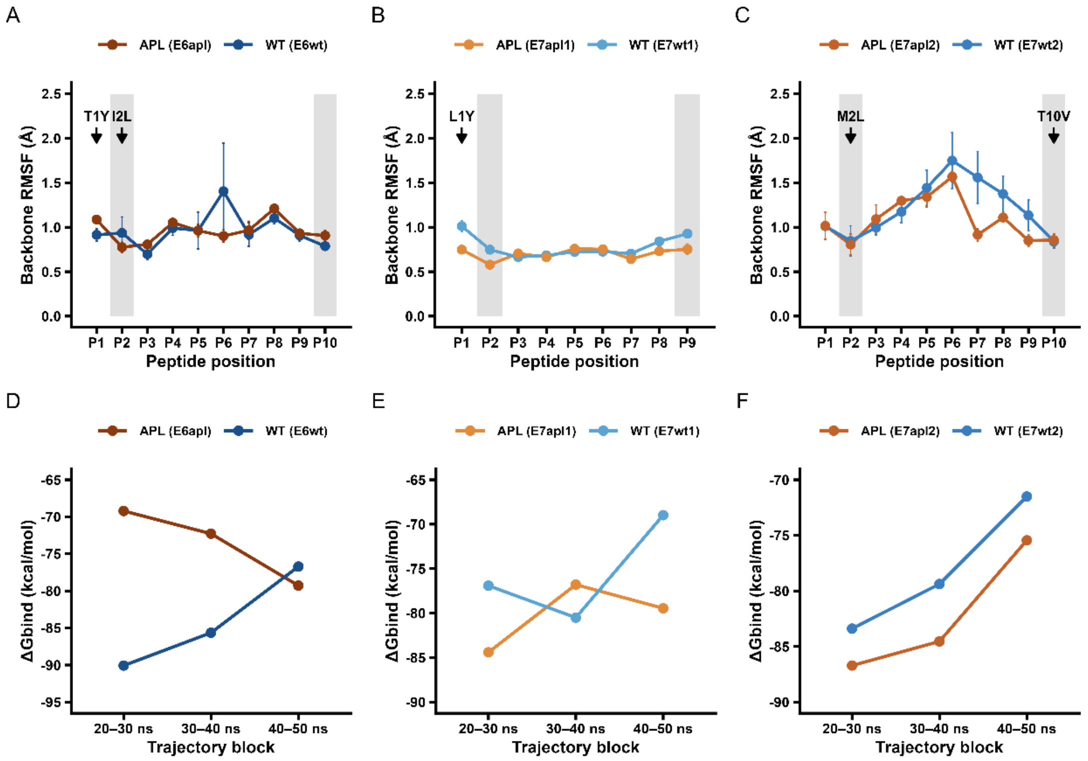

Figure S5. IEDB-based population coverage analysis of the selected HLA-A*02:01-restricted HPV16 epitopes.

Population coverage analysis was performed using the Immune Epitope Database and Analysis Resource (IEDB) Population Coverage tool based on the selected HLA-A*02:01-restricted HPV16 E6/E7 epitopes. The predicted class I population coverage was evaluated in different geographic populations, including **(A) Worldwide, (B) East Asia, (C) Europe, and (D) North America**. The bar plots represent the percentage distribution of individuals according to the number of recognized epitope hits/HLA combinations, while the curves indicate the cumulative population coverage. The analysis provides an estimation of the potential population applicability of the selected HLA-A*02:01-restricted epitopes.

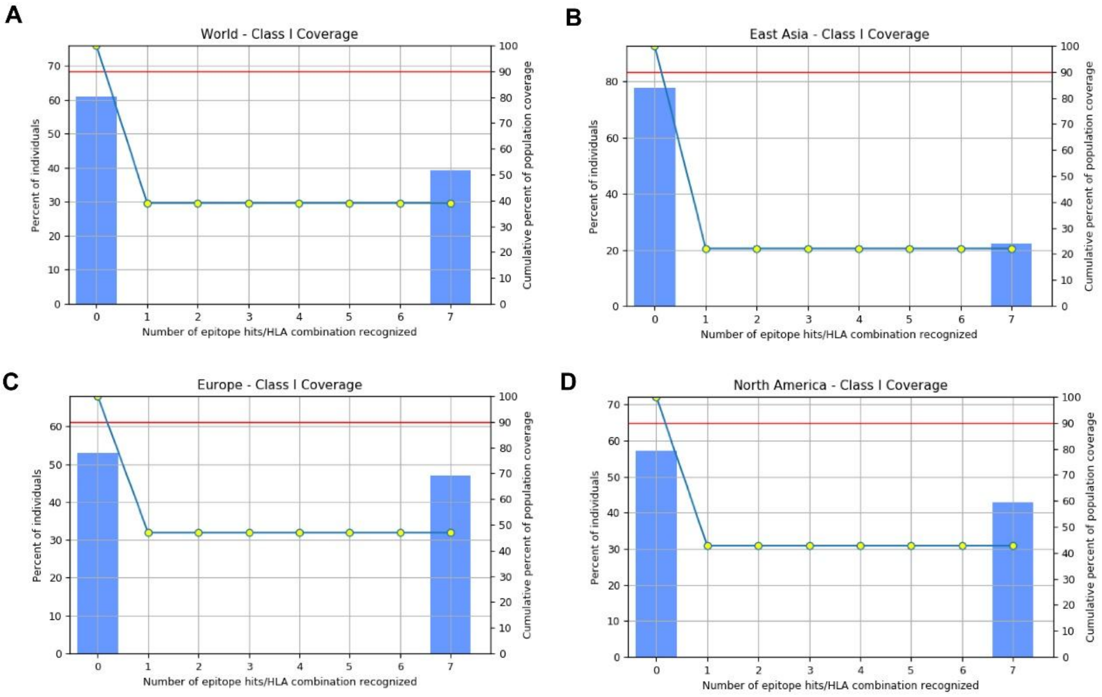

Table S1. Conservation of HPV16 residues targeted for altered peptide ligand (APL) optimization.

The conservation of wild-type residues corresponding to APL substitution sites was evaluated across 16 representative HPV16 sublineages. All targeted residues showed complete conservation (100%), indicating that APL substitutions were introduced at highly conserved viral positions rather than naturally variable sites.

APL candidate	Substitution	WT residue	Introduced residue	Dominant natural residue	Conservation (%)
E6apl	T→Y	T	Y	T	100.0
E6apl	I→L	I	L	I	100.0
E7apl1	L→Y	L	Y	L	100.0
E7apl2	T→V	T	V	T	100.0
E7apl3	M→L	M	L	M	100.0
E7apl3	T→V	T	V	T	100.0

Table S2. Overview of peptide evaluation, experimental validation, and final formulation selection.

Peptide	Source epitope	In silico prediction	T2 stabilization	CD8+ T-cell expansion	IFN-γ ELISpot	MD simulation	Final formulation
E6wt	E6 29–38	✓	✓	✓	✓	✓	✓
E6apl	E6 29–38	✓	✓	✓	✓	✓	✓
E7wt1	E7 82–90	✓	Not tested*	✓	✓	✓	✓
E7apl1	E7 82–90	✓	Not tested*	✓	✓	✓	No
E7wt2	E7 11–20	✓	✓	✓	✓	✓	✓
E7apl2	E7 11–20	✓	✓	✓	✓	✓	✓
E7apl3	E7 11–20	✓	✓	✓	✓	No	✓

Note: E7wt1 and E7apl1 were not evaluated in the T2 stabilization assay due to peptide-related solubility/DMSO-associated cytotoxicity issues. E7apl1 was excluded from the final formulation because it showed reduced functional activity despite improved predicted HLA-A*02:01 binding. E7apl3 was included based on superior functional performance but was not subjected to MD simulation.

Table S3. Summary of IEDB population coverage analysis for the selected HLA-A*02:01-restricted HPV16 epitopes.

Population	Coverage (%)	Average hit	PC90
Worldwide	39.08	2.74	1.15
East Asia	22.20	1.55	0.90
Europe	47.00	3.29	1.32
North America	42.82	3.00	1.22

Note: Population coverage was predicted using the IEDB Population Coverage tool based on the selected HLA-A*02:01-restricted HPV16 E6/E7 epitopes. Coverage (%) represents the estimated percentage of individuals predicted to recognize at least one epitope-HLA combination. Average hit indicates the average number of recognized epitope-HLA combinations per individual. PC90 represents the minimum number of epitope-HLA combinations required to achieve 90% population coverage.
