# Supplementary material for: Immunoinformatics-Driven Rational Design of Altered Peptide Ligands and In Vitro Validation of a Multi-Epitope CTL Formulation Targeting HPV16 E6/E7
Source: Vaccines (Basel). 2026 Aug 20;14(8):715. doi: 10.3390/vaccines14080715 (PMC13517650; doi:10.3390/vaccines14080715)
Supplement: Supplementary file 1 [file vaccines-14-00715-s001.zip › Table S1. Conservation of HPV16 residues targeted for altered peptide ligand (APL) optimization.pdf]

Table S1. Conservation of HPV16 residues targeted for altered peptide ligand (APL) optimization.

The conservation of wild-type residues corresponding to APL substitution sites was evaluated across 16 representative HPV16 sublineages. All targeted residues showed complete conservation (100%), indicating that APL substitutions were introduced at highly conserved viral positions rather than naturally variable sites.

APL candidate	Substitution	WT residue	Introduced residue	Dominant natural residue	Conservation (%)
E6apl	T→Y	T	Y	T	100.0
E6apl	I→L	I	L	I	100.0
E7apl1	L→Y	L	Y	L	100.0
E7apl2	T→V	T	V	T	100.0
E7apl3	M→L	M	L	M	100.0
E7apl3	T→V	T	V	T	100.0
