# Supplementary material for: Immunoinformatics-Driven Rational Design of Altered Peptide Ligands and In Vitro Validation of a Multi-Epitope CTL Formulation Targeting HPV16 E6/E7
Source: Vaccines (Basel). 2026 Aug 20;14(8):715. doi: 10.3390/vaccines14080715 (PMC13517650; doi:10.3390/vaccines14080715)
Supplement: Supplementary file 1 [file vaccines-14-00715-s001.zip › Table S2. Overview of peptide evaluation, experimental validation, and final formulation selection.pdf]

Table S2. Overview of peptide prioritization, experimental evaluation, and final formulation selection..

Peptide	Source epitope	In silico prediction	T2 stabilization	CD8+ T-cell expansion	IFN-γ ELISpot	MD simulation	Final formulation
E6wt	E6 29–38	✓	✓	✓	✓	✓	✓
E6apl	E6 29–38	✓	✓	✓	✓	✓	✓
E7wt1	E7 82–90	✓	Not tested*	✓	✓	✓	✓
E7apl1	E7 82–90	✓	Not tested*	✓	✓	✓	No
E7wt2	E7 11–20	✓	✓	✓	✓	✓	✓
E7apl2	E7 11–20	✓	✓	✓	✓	✓	✓
E7apl3	E7 11–20	✓	✓	✓	✓	Not performed	✓

Note: E7wt1 and E7apl1 were not evaluated in the T2 stabilization assay due to peptide-related solubility/DMSO-associated cytotoxicity issues. E7apl1 was excluded from the final formulation because it showed reduced functional activity despite improved predicted HLA-A*02:01 binding. E7apl3 was included based on superior functional performance but was not subjected to MD simulation.
