# Supplementary material for: Immunoinformatics-Driven Rational Design of Altered Peptide Ligands and In Vitro Validation of a Multi-Epitope CTL Formulation Targeting HPV16 E6/E7
Source: Vaccines (Basel). 2026 Aug 20;14(8):715. doi: 10.3390/vaccines14080715 (PMC13517650; doi:10.3390/vaccines14080715)
Supplement: Supplementary file 1 [file vaccines-14-00715-s001.zip › Table S3. Summary of IEDB population coverage analysis for the selected HLA-A0201-restricted HPV16 epitopes..pdf]

Table S3. Summary of IEDB population coverage analysis for the selected HLA-A*02:01-restricted HPV16 epitopes.

Population	Coverage (%)	Average hit	PC90
Worldwide	39.08	2.74	1.15
East Asia	22.20	1.55	0.90
Europe	47.00	3.29	1.32
North America	42.82	3.00	1.22
